# An overview of the SAMPL8 host–guest binding challenge

**DOI:** 10.1007/s10822-022-00462-5

**Published:** 2022-10-14

**Authors:** Martin Amezcua, Jeffry Setiadi, Yunhui Ge, David L. Mobley

**Affiliations:** 1 Department of Pharmaceutical Sciences, University of California, Irvine, CA 92697 USA; 2 Department of Chemistry, University of California, Irvine, CA 92697 USA; 3 Skaggs School of Pharmacy and Pharmaceutical Sciences, University of California, San Diego, La Jolla, CA, 92093 USA

**Keywords:** Host–guest binding, Free energy, Binding affinity, SAMPL, Blind challenge, Octaacid, Cucurbituril

## Abstract

The SAMPL series of challenges aim to focus the community on specific modeling challenges, while testing and hopefully driving progress of computational methods to help guide pharmaceutical drug discovery. In this study, we report on the results of the SAMPL8 host–guest blind challenge for predicting absolute binding affinities. SAMPL8 focused on two host–guest datasets, one involving the cucurbituril CB8 (with a series of common drugs of abuse) and another involving two different Gibb deep-cavity cavitands. The latter dataset involved a previously featured deep cavity cavitand (TEMOA) as well as a new variant (TEETOA), both binding to a series of relatively rigid fragment-like guests. Challenge participants employed a reasonably wide variety of methods, though many of these were based on molecular simulations, and predictive accuracy was mixed. As in some previous SAMPL iterations (SAMPL6 and SAMPL7), we found that one approach to achieve greater accuracy was to apply empirical corrections to the binding free energy predictions, taking advantage of prior data on binding to these hosts. Another approach which performed well was a hybrid MD-based approach with reweighting to a force matched QM potential. In the cavitand challenge, an alchemical method using the AMOEBA-polarizable force field achieved the best success with RMSE less than 1 kcal/mol, while another alchemical approach (*ATM/GAFF2-AM1BCC/TIP3P/HREM*) had RMSE less than 1.75 kcal/mol. The work discussed here also highlights several important lessons; for example, retrospective studies of reference calculations demonstrate the sensitivity of predicted binding free energies to ethyl group sampling and/or guest starting pose, providing guidance to help improve future studies on these systems.

**Supplementary Information:**

The online version contains supplementary material available at 10.1007/s10822-022-00462-5.

## Introduction

Quantitative modeling done with molecular simulations can be used to estimate thermodynamic and/or physical properties, with the goal of aiding and directing small molecule drug design for therapeutic development [4–8]. Simulation-based binding free energy calculations have gained much attention for their potential to help accelerate early-stage drug discovery [9]. The accuracy of free energy calculations depends on and is commonly limited by the degree of accuracy of the force field [10–12], sampling [13–17], and how the system is set up (i.e., protonation state, chosen tautomer state, buffer concentration, etc.) [18, 19].

For well-behaved protein–ligand systems, free energy methods can achieve agreement with experiments within about 1–2 kcal/mol [20–22]. However, protein–ligand systems are not always well-behaved because of the highly dynamic nature of proteins, where conformational dynamics can frequently be in the microsecond to millisecond timescale or slower, thwarting computation of true equilibrium binding free energies [20, 23]. In such cases, it is difficult to assess how much of the inaccuracy is due to limitations in a chosen force field versus sampling limitations [20, 24], and sometimes other factors. However, even when using well-behaved protein–ligand systems that are free of slow motions, other factors such as ionizable residues that change protonation state upon ligand binding can complicate assessment of computational methods.

### Host–guest systems: What are they? Why use them?

Supramolecular host–guest complexes have been used as simpler surrogate binding systems, instead of protein–ligand systems, to assess modeling errors and test computational methods and force fields. Host–guest systems feature hosts, or ”mini-receptors”, which are smaller and often more rigid, hence eliminating some challenges associated with the modeling of proteins. Since hosts are smaller (typically less than 100 non-hydrogen atoms) simulations can be run quicker and longer. These hosts can bind guest molecules (in some cases, small drug-like compounds) with protein–ligand-like affinities [20, 25, 26]. In addition, hosts may undergo conformational changes upon binding, have hydrophilic and hydrophobic interactions, and protonation states are often predictable with high confidence [27]. However, host–guest systems still involve some complexities in molecular recognition. These properties and characteristics have made host–guest systems a popular model to help identify limitations and deficiencies in force fields and/or methods [10, 18, 28–30].

### SAMPL challenges: history, purpose, and direction

SAMPL (Statistical Assessment of the Modeling of Proteins and Ligands) is an NIH-funded project consisting of a series of blind crowdsourcing challenges which serve to test and improve computational methods as reliable predictive tools for rational drug design [11, 18, 20, 28, 30, 31]. Since its inception in 2008, SAMPL has featured predictions of physical properties of drug-like small molecules as well as binding free energies for host–guest systems, as well as occasional protein–ligand challenges.

Over the course of SAMPL, by focusing the community on specific modeling difficulties with well designed systems, host–guest challenges have driven progress in many areas and advanced our understanding of sources of error [11, 18–20, 28–30, 32–46]. SAMPL has helped focus attention on the effects of co-solvents and ions in modulating binding (these effects, when neglected, result in errors of up to 5 kcal/mol [20]), and the importance of adequately sampling water rearrangements [18, 20, 24].

Although host–guest systems are ”simpler”, they still pose several modeling difficulties. For example, guests bearing a formal charge can be especially difficult to treat. Indeed, charged guests were shown to affect the accuracy of many methods in SAMPL7 [11]. Polarization potentially plays a big role in predictive accuracy when modeling systems with charged molecules and in the presence of explicit water. A polarizable model used in the last two SAMPL iterations has outperformed all other methods in the datasets for which predictions were made. In SAMPL7 we observed that across all host–guest systems, methods using this polarizable force field approach provided additional accuracy [11, 44] but with an increase in computational cost due to the added complexity of the physical model to describe the systems. On the other hand, we have seen several methods using fixed charge force fields with performances comparable to the more expensive polarizable models. But these methods contain empirical corrections that rely on adjusting predictions for the particular target based on prior studies of that target [11, 20, 45]. This would not be ideal for new datasets and in real world applications where there is little or no data available on a target.

Several SAMPL iterations have helped identify obstacles that need to be addressed and further studied, and are described in the literature [11, 18–20, 24, 30]. While work continues to seek to address these difficulties, new innovations have not always led to clear conclusions. In some cases, performance of a given method remains variable across several challenges. Particularly, performance has been highly variable by method and target, and until recently no method had emerged as universally reliable across all systems or most systems. In SAMPL7, several methods showed reasonable accuracy (RMSE under 3 kcal/mol) across multiple hosts, and one method had RMSE under 2 kcal/mol. In SAMPL8, we hope to see methods which perform reliably across multiple hosts, and determine whether any method(s) improve relative to prior challenges.

### SAMPL8 host–guest Systems

SAMPL host–guest datasets involve hosts such as cyclodextrins [47, 48], cucurbiturils and cucurbituril-like [49, 50], and Gibb deep cavity cavitands, GDCCs [51–54], with drug-like small molecules or fragments. Various hosts in these families have been studied or benchmarked in SAMPL [11, 18, 20, 30] and elsewhere [24], and provide insights on particular difficulties within each system. One common theme we observe after several iterations of SAMPL is that predictions for cavitands are typically more accurate, while cucurbiturils and cucurbituril-like hosts (including clips) are more challenging.

Key modeling difficulties for the hosts studied in SAMPL8 have been highlighted previously as noted above, and are discussed here briefly. Host binding sites with tight entry portals can have barriers preventing entry or exit of guests with bulky cores. This can limit and hinder sampling of guests and lead to convergence problems. Ensuring adequate host conformational sampling and guest sampling is needed for accurate binding free energy calculations. Slow motion of waters into and out of the cavity, with the number of water fluctuations occurring at timescales of over tens of nanoseconds has been shown to affect binding free energy predictions [55]. The slow fluctuation of waters is thought to occur in the absence of strong binders. In addition, salt concentration and the buffer conditions may modulate binding, the hosts may bind ions which can compete with other ligands for the binding site, and affect the accuracy of the binding affinity predictions. Charged guests can pose methodological challenges and may introduce finite-size artifacts that need to be accounted for [12]. The protonation states of the host and/or guest may be modified upon binding, and if there is a significant p*K*_a_shift of titratable groups, treating the wrong protonation state (or even only a single protonation state) may lead to large errors in binding free energy estimates [56].

Three hosts in the GDCC and cucurbit[n]uril (CB[n]) families were chosen for the SAMPL8 host–guest challenge. We aimed to study modeling implication(s) of tetra-endo-methyl Octa-acid (TEMOA) and a variant tetra-endo-ethyl Octa-acid (TEETOA), with flexible ethyl side-chains in the presence of mostly rigid guests. In addition, SAMPL8 also revisits the cucurbit[8]uril (CB8) host, with a series of guests which are addictive and commonly abused drugs [57].

#### CB8: drugs of abuse challenge

**Fig. 1:**
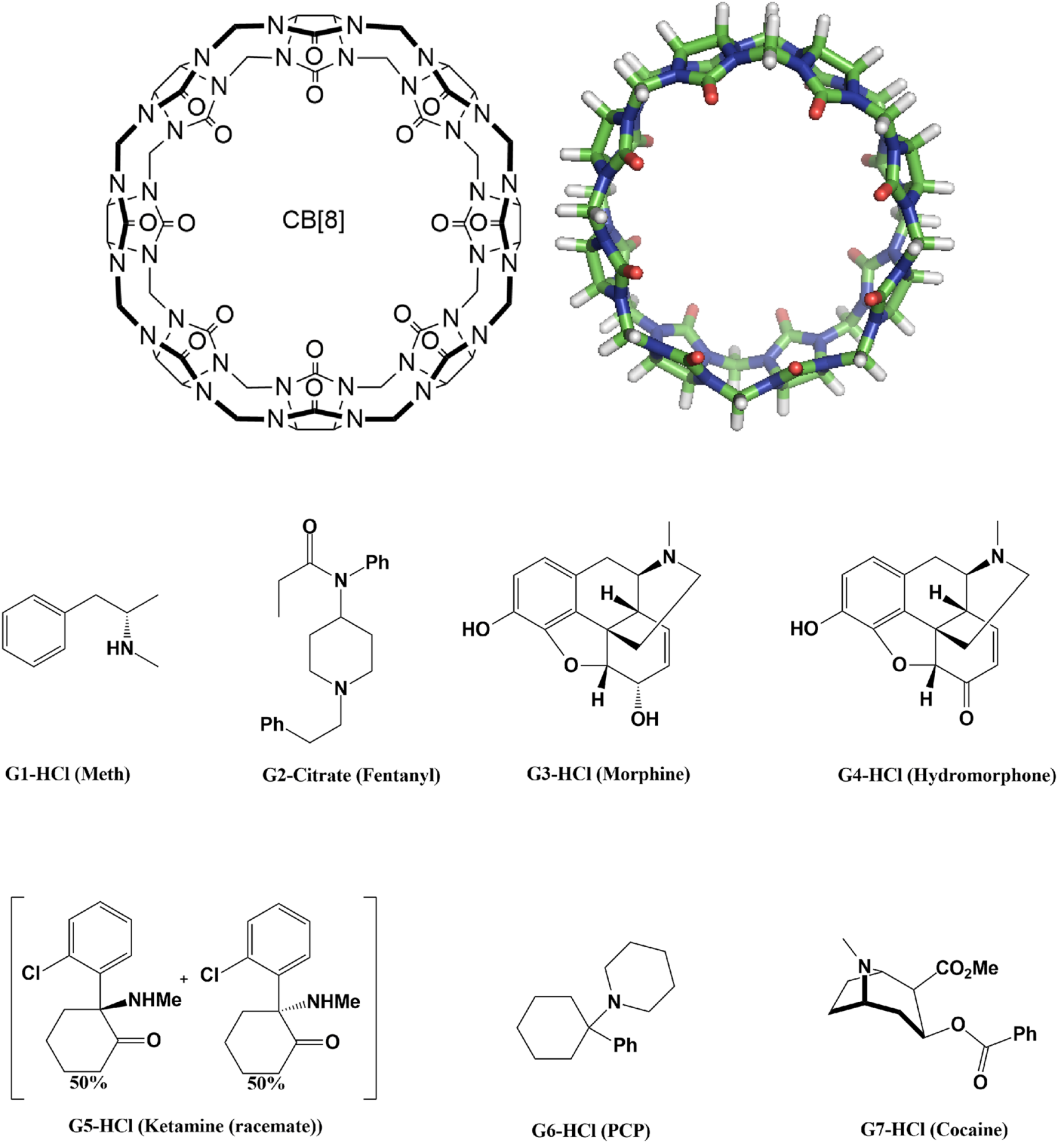
Structures of the CB8 host and drugs of abuse as guests molecules for SAMPL8. The barrel shaped macrocycle, CB8, host is shown on the top. It is composed of eight glycoluril units, and its carbonyl portal interacts and binds with cationic ammonium based guests inside the cavity. The guests for the SAMPL8 challenge are drugs of abuse (methamphetamine, fentanyl, morphine, hydromorphine, ketamine, PCP, and cocaine) which have the characteristics of typical CB[n] binders. The guests are named G1 through G7

The first dataset developed was for the CB8 host (Fig. 1). The host was previously featured in SAMPL3 [28] and SAMPL6 [20], and is similar to other cucurbituril analogs such as CB7, CB-Clip [58], and TrimerTrip [11, 49]. The CB8 “drugs of abuse” challenge focuses on binding of CB8 to nine guests which are drugs of abuse, including morphine, hydromorphone, methamphetamine, cocaine, and others (Fig. 1). The list of guests on GitHub also includes cycloheptanamine and cyclooctanamine (G8 and G9); however, these were not part of the challenge since their experimental values were previously reported. Experimental binding affinities were measured by competition with these guests.

CB[n]–guest complexes are well known to have very high affinity, especially for cationic ammonium and diammonium guests like those featured in SAMPL8. The high affinity measurements from these systems have been attributed to an enthalpic driving force provided by the lack of host hydrogen bonds with intracavity waters [59]. Upon binding, protons of the guest’s nitrogen interact with the oxygen of the CB8 carbonyl portal, which limits the number of poses that need to be considered computationally [41].

Previous studies on cucurbiturils have provided some highlights and important factors to consider [20, 30, 60]. First, we note that since CB8 is fairly rigid, sampling of the host may be straightforward. However, cucurbiturils and other SAMPL hosts have been observed to collapse in on themselves with certain force fields [18, 61], which is thought to limit guest sampling, affect convergence, and result in overestimated free energies. Second, guest binding modes have been shown to be more challenging to adequately sample [20, 62, 63], especially when the guest is more flexible. Perhaps more relevant to this dataset is that cucurbiturils are known to modulate protonation states of guests upon binding [64, 65].

For this challenge we hoped participants would submit multiple methods which varied by only changing a single simulation parameter, such as a force field or an aspect of the simulation protocol, since such variations would allow us to directly probe the sensitivity of results to particular choices. Thus for our reference calculations the goal was to compare and test different force fields. At the same time, several protonation states needed to be considered for one guest. In particular, ketamine (G5), since its p*K*_a_value was near the experimental pH of 7.4. However, it was possible that alternative protonation states were accessible for other guests, thus necessitating their consideration. In such cases, close attention to the geometry of certain trivalent nitrogen centers would be required because if protonated, computationally they can act as a chiral center and all guest geometries might need to be sampled. If geometric sampling is inadequate, the selected geometry may impact the binding estimates for some methods.

#### GDCCs (Gibb deep cavity cavitands): sterics and flexibility challenge

**Fig. 2:**
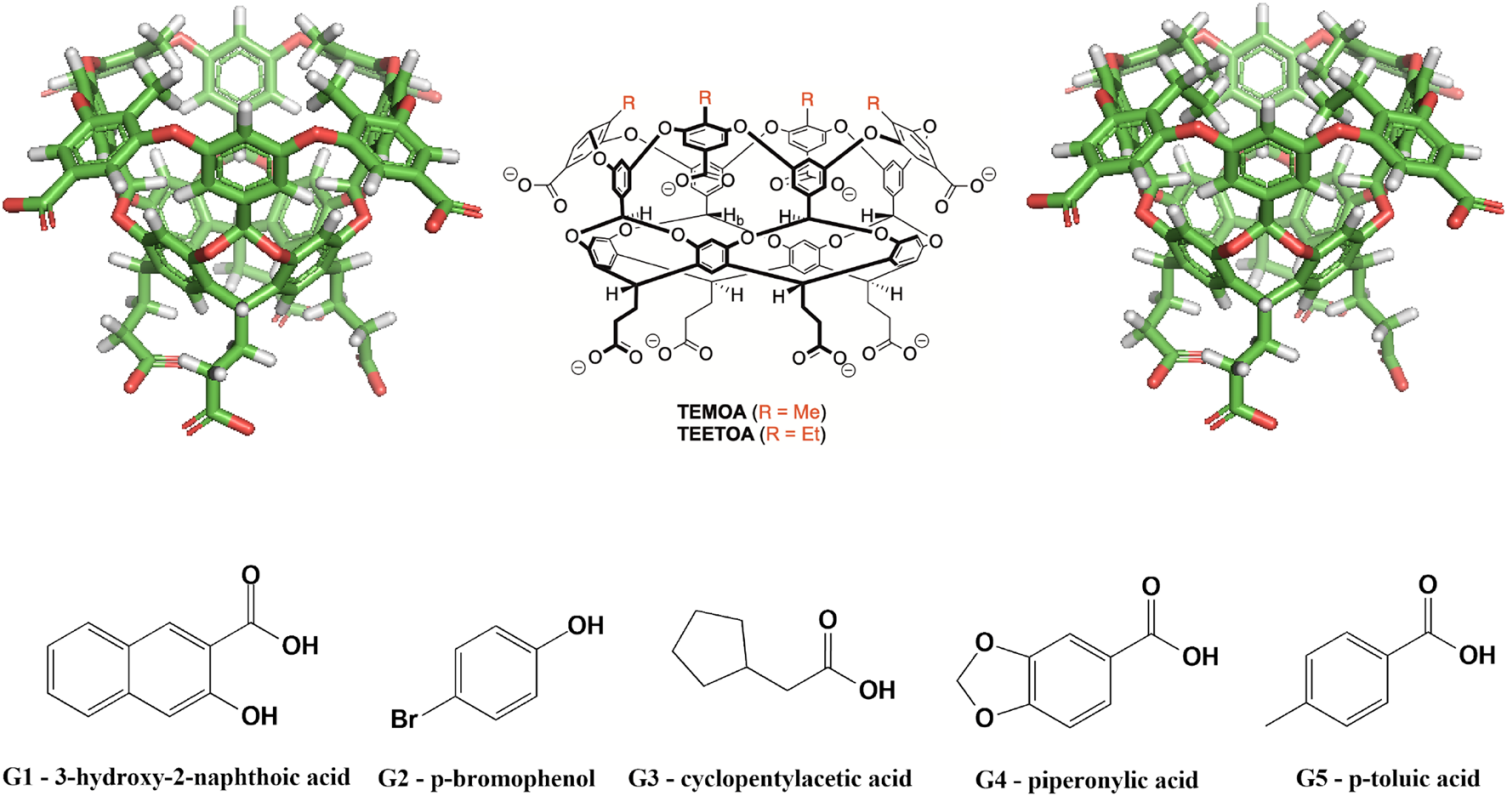
Structures of the GDCC hosts and guest molecules for the SAMPL8. (Top) TEMOA and TEETOA hosts in 2D and 3D representation. The 3D ball and stick figures are of TEMOA on the left and TEETOA on the right, (bottom) guests G1–5. The difference between the hosts is the functionality near the cavity opening, TEMOA has four methyl groups while TEETOA has four ethyl groups. The guests for SAMPL8 are named G1–5. Guests are fairly rigid molecules with carboxylate and/or hydroxyl groups. For TEMOA–G3, binding free energies were previously reported, thus was made optional for participants

The second dataset featured two hosts in the GDCC family, TEMOA and TEETOA, commonly referred to as Octa-Acids. GDCCs are low-symmetry hosts, fairly rigid, and have a basket-shaped binding site with eight carboxylate groups appended to the host to enhance solubility [24, 66]. Four carboxylates are located near the cavity protruding out to solvent, and four others are at the bottom of the host at the propionate tails. TEMOA has been used in previous SAMPL host–guest challenges [18, 20, 30] with different sets of guests, and appeared with the name OAMe. TEETOA is a new variant synthesized by the Gibb lab, and differs from TEMOA by four ethyl groups which reduce the size of the cavity entry, may elongate the cavity entry, and introduce flexibility at the entrance (Fig. 2). The guests selected for both TEMOA and TEETOA are mostly rigid with a hydrophobic moiety and a polar region at opposite ends of the molecules. The hydrophilic region of the guests are composed of carboxylate and/or hydroxyl groups (Fig. 2), and when the guests are in complex with the host, the polar group is typically exposed to the solvent while the hydrophobic region is buried in the deep hydrophobic binding site.

In this study we employ the attach–pull–release (APR) methodology, using the pAPRika toolkit [67], for the SAMPL8 host–guest blind challenge to predict absolute binding free energies as a reference (described in detail in Sect. 2.4). We selected this approach because it has been used with considerable success to study host–guest binding in several host–guest systems previously, and provides relatively high throughput binding free energy calculations in our hands. We also evaluate the performance of other methodologies submitted by participants from various academic institutions and/or industry. The remainder of the paper is organized as follows: we provide a general description of the types of methods submitted for this challenge, give detail on the method(s) used for reference calculations, and finally go through the results, lessons learned, and conclusions.

## Methods

In this section we give challenge organization details, a general overview of methods used by participants’ for their submissions, describe the details of reference calculations, summarize experimental details and methodologies (experimental studies are published separately [57]), and describe the statistical analysis and evaluate approaches.

### Challenge organization

The SAMPL8 host–guest challenge was organized similar to SAMPL7, allowing participants to submit their “top” predictions as a ranked submission for any or all of the datasets (CB8 and GDCC), but a submission for either dataset was required to include all guests. Only ranked submissions were considered for the main analysis of the challenge. Additional submissions were allowed, but needed to be denoted as non-ranked. Non-ranked submissions gave us an opportunity for benchmarking and provided additional methods for cross comparison, while still allowing each participant only one opportunity to formally compete in the challenge. All participants formally submitted blind predictions prior to the challenge deadline. In addition, two members of our team (MA and JS) conducted blind reference calculations which were submitted informally in the non-ranked category.

Pre-prepared host and guest structure files (as MOL2, PDB, and SDF) and SMILES strings were provided to participants. Where applicable, host and/or guest structures were provided with reasonable protonation states. However, we advised participants to exercise caution in the choice of protonation state, particularly for CB8–G5, TEMOA–G2, and TEETOA-G2. All files described above, data, and instructions are available on the SAMPL8 GitHub repository (https://github.com/samplchallenges/SAMPL8/tree/master/host_guest).

A prescribed submission template was provided for participants to follow and included an example submission with all needed information. Participants were required to follow the template, since we would use automated scripts to parse and run the statistical analysis. Each submission required: predictions, participant name, participant organization, name of their method, list of used software, detailed method description, method category, and a ranked or non-ranked classification. In the predictions section, we required a predicted free-energy, free-energy SEM, and free energy model uncertainty. Predicted binding enthalpy values were optional. Participant and reference calculation submission files are available in relevant host submission directory in the SAMPL8 GitHub repository (https://github.com/samplchallenges/SAMPL8/tree/master/host_guest/Analysis/Submissions).

Data collection was finalized around June 9, 2020 for the CB8 drugs of abuse challenge and October 14, 2020 for the GDCC sterics and flexibility challenge. Submission deadlines were set for September 15, 2020 and February 21, 2021 (updated from February 4, 2021), respectively. Submissions for CB8–G8, CB8–G9, and TEMOA–G3 were optional since these binding values have been reported in literature.

### Statistical analysis of challenge submissions

The statistical analysis of the challenge was performed using Python scripts adopted from SAMPL6 and SAMPL7, and deposited in the SAMPL8 GitHub repository. Thus, for SAMPL8 we use statistical metrics as used in previous SAMPL iterations [11, 18, 20] which include RMSE (root mean-squared error), $R^{2}$ (coefficient of determination), $\tau$ (Kendall Tau correlation coefficient), m (linear regression slope), ME (mean error) and MAE (mean absolute error). Bootstrapping with replacement was used to determine uncertainty in the error metrics as described in the literature [18, 20]. In addition, the RMSE, MAE, and ME of each individual host–guest system (considering all methods) were used to identify and compare the most accurate and least accurate predicted systems.

The statistical analysis was separated into ranked and non-ranked categories. For the non-ranked category, all submissions (ranked and non-ranked) were considered, while in ranked category (for formal competition) only ranked submissions were considered. For datasets with optional systems, analysis was done with and without those guests, where the latter was denoted as “no optional” in the respective directories. All data, plots, and tables for ranked and non-ranked analysis subsets are available in the SAMPL8 GitHub repository (https://github.com/samplchallenges/SAMPL8/tree/master/host_guest/Analysis).

### Participant methodologies

For SAMPL8, many methods used alchemical free energy calculations with classical fixed charge force fields (GAFF [68] or GAFF2, CGenFF [69, 70]), explicit water models (TIP3P [71], TIP4PEw [72, 73]), and with a AM1-BCC [74, 75] or RESP [76] charging scheme. One method utilized the polarizable force field AMOEBA [3, 77], while other approaches used force matching [78] starting from CGenFF parameters. Apart from simulation-based free energy methods, other approaches included quantum mechanics (QM) and QM/MM (molecular mechanics) and machine learning. A summary of the groups methodologies are described in Table 1 and in more detail in the relevant literature.

**Table 1:** Summary of methods (ranked and non-ranked) used in the SAMPL8 host–guest challenge for binding free energy calculations

ID	Sid	Energy model	Solvent model	Sampling	Ranked	SAMPL8 References
CB8						
*SILCS/LGFE/TIP3P/GCMC-MD/rew* (C)	5	CGenFF	TIP3P (E)	GCMC-MD	No	
*DDM/FEP/MBAR/FM/RW/[pm6s6]*	28	FM	FM (E)	MD	Yes	[79]
*ML/NNET/CORINA-descriptors2*	10	xtb-GFN2B	–*	–*	No	
*SILCS/LGFE/TIP3P/GCMC-MD*	3	CGenFF	TIP3P (E)	GCMC-MD	Yes	
*SILCS/LGFE/TIP3P/GCMC-MD/G5*	4	CGenFF	TIP3P (E)	GCMC-MD	No	
*DDM/FEP/MBAR/Paramchem/FM/[gfn2gfn2]*	19	FM-XTB	TIP3P (E)	MD	No	[79]
*ML/NNET/CORINA-descriptors1*	9	xtb-GFN2B	–*	–*	No	
*DDM/FEP/MBAR/FM/[pm6s6]*	22	FM-PM6-D3H4	TIP3P (E)	MD	No	[79]
*DDM/FEP/MBAR/FM/RW/[wb97xd,s6]*	29	FM-B3LYP(H), FM-WB97X-D(G)	TIP3P (E)	MD	No	[79]
*DDM/FEP/MBAR/Paramchem/FM/[C36S6]*	18	FM-B3LYP (H), Paramchem-C36 (G)	TIP3P (E)	MD	No	[79]
*DDM/FEP/MBAR/FM/[mp2,b3lyp]*	15	FM-B3LYP(H), FM-MP2 (G)	TIP3P (E)	MD	Yes	
*DDM-SAMS/GAFF-DMBIS/TIP3P/MCMC-SAMS/*	2	GAFF-DMBIS(B3LYP)	TIP3P (E)	MCMC-SAMS	Yes	
APR/GAFF2-AM1BCC/TIP3P/US/TI**	33	GAFF2	TIP3P (E)	MD-US	No	
APR/GAFF2-AM1BCC/TIP3P/US/MBAR**	34	GAFF2	TIP3P (E)	MD-US	No	
*DDM/FEP/MBAR/Paramchem/FM/RW/[wb97xd]*	26	FM-B3LYP(H), FM-WB97X-D(G)	TIP3P (E)	MD	No	[79]
*GFN2-xTB/MetaMD/GBSA/ensemble/Nobuffer*	1	xtb-GFN2	GBSA (I)	MTD	Yes	
APR/OPENFF1.2.0-AM1BCC/TIP3P/US/TI**	31	Parsley-AM1BCC	TIP3P (E)	MD-US	No	
APR/OPENFF1.2.0-AM1BCC/TIP3P/US/MBAR**	32	Parsley-AM1BCC	TIP3P (E)	MD-US	No	
*US/GAFF-AM1BCC/TIP3P/HRE-MD/emp_corr* (C)	11	GAFF	TIP3P (E)	HRE-MD	Yes	[80]
*LiGaMD/q4MD/TIP4P/enhanced-sampling*	16	q4MD-RESP	TIP4P (E)	Ga-MD	No	
*DDM/FEP/MBAR/C36*	17	Paramchem-C36	TIP3P (E)	MD	No	[79]
*GAFF-RESP/TIP3P/MD/xtb-GFN2B/Bolts-Avg*	8	GAFF-RESP	TIP3P (E)	MD	No	
*GAFF-RESP/TIP3P/MD-Classical/xtb-GFN2B*	7	GAFF-RESP	TIP3P (E)	MD	No	
*DDM/FEP/MBAR/FM/[mp2s6]*	21	FM-B3LYP(H), FM-MP2(G)	TIP3P (E)	MD	No	[79]
*MD/fmB3LYP(H)-fmMP2(G)/TIP3P/REUS/*	13	FM-B3LYP (H), FM-MP2 (G)	TIP3P (E)	REUS	Yes	[79]
*DDM/FEP/MBAR/FM/[gfn2,s6]*	23	FM-B3LYP(H), xtb-GFN2(G)	FM (E)	MD	No	[79]
*DDM/FEP/MBAR/FM/RW/[blyp,s6]*	24	FM-B3LYP(H), FM-WB97X-D(G)	TIP3P (E)	MD	No	[79]
*DDM/FEP/MBAR/FM/[pm6pm6]*	20	FM-PM6-D3H4	TIP3P (E)	MD	No	[79]
*DDM/FEP/MBAR/FM/RW/[blyp,s6BLUR]*	25	FM-B3LYP-BLUR(H), FM-WB97X-D(G)	TIP3P (E)	MD	No	[79]
*ABFE/Parsley-GAFF-BCC/TIP3P/MD/NoBuffer2*	14	Parsley-AM1BCC	TIP3P (E)	MD	No	
*ABFE/Parsley-GAFF-BCC/TIP3P/MD/NoBuffer1*	30	Parsley-AM1BCC	TIP3P (E)	MD	Yes	
*DDM/FEP/MBAR/FM/RW/[wb97xdBLUR]*	27	FM-B3LYP(H), FM-WB97X-D/DEF2-SVP-BLUR(g)	TIP3P (E)	MD	No	[79]
*EE-MCC/GAFF2-AM1BCC/TIP3P/MD/*	6	GAFF2-AM1BCC	TIP3P (E)	MD	Yes	[81]
*US/GAFF-AM1BCC/TIP3P/HRE-MD*	12	GAFF-AM1BCC	TIP3P (E)	HRE-MD	No	[80]
GDCC–TEMOA and TEETOA						
*DDM/AMOEBA/BAR*	44	AMOEBA	AMOEBA (E)	MD	Yes	
*ATM/GAFF2-AM1BCC/TIP3P/HREM*	37	GAFF2-AM1BCC	TIP3P (E)	HRE	Yes	[82]
*PMF/GAFF2-AM1BCC/TIP3P/MD-US*	38	GAFF2-AM1BCC	TIP3P (E)	MD-US	Yes	[82]
*AM1BCC/MMPBSA/TIP4PEW/MD_NR3*	42	GAFF2-AM1BCC	TIP4PEW (E)	MD	No	
*AM1BCC/MMPBSA/TIP4PEW/MD*	43	GAFF2-AM1BCC	TIP4PEW (E)	MD	Yes	
*AM1BCC/MMPBSA/TIP4PEW/MD_NR2*	41	GAFF2-AM1BCC	TIP4PEW (E)	MD	No	
*ML/NNET/CORINA-descriptors-8*	39	xtb-GFN2B	–*	–*	Yes	
*SILCS/LGFE/TIP3P/GCMC-MD*	36	GAFF	TIP3P (E)	GCMC-MD	Yes	
*SILCS/LGFE/TIP3P/GCMC-MD_NR*	35	GAFF	TIP3P (E)	GCMC-MD	No	
APR/OPENFF1.2.0-AM1BCC/TIP3P/US/TI**	48	Parsley-AM1BCC	TIP3P (E)	US	No	
APR/OPENFF1.2.0-AM1BCC/TIP3P/US/MBAR**	49	Parsley-AM1BCC	TIP3P (E)	US	No	
APR/GAFF2-AM1BCC/TIP3P/US/MBAR**	51	GAFF2-AM1BCC	TIP3P (E)	US	No	
APR/GAFF2-AM1BCC/TIP3P/US/TI**	50	GAFF2-AM1BCC	TIP3P (E)	US	No	
*DDM/C36/TIP3P/MD/MBAR*	45	Paramchem-C36	TIP3P (E)	MD	Yes	
*LiGaMD/GAFF2/RESP/TIP4P/Sampling*	47	q4MD-RESP	TIP4P(E)	GaMD	No	
*MD/ParamChem/TIP3P/REUS/*	46	Paramchem-C36	TIP3P (E)	REUS	Yes	
*AM1BCC/MMPBSA/TIP4PEW/MD_NR1*	40	GAFF2-AM1BCC	TIP4PEW (E)	MD	No	

The use of explicit and/or implicit solvents is flagged by an (E) or (I) respectively, and a correction approach was taken on methods flagged with a (C). If the host (H) and guest (G) are parametrized with a different energy model they will be flagged respectively. Where a machine learning approach was taken, certain categories are not relevant and flagged with an asterisk (*). Reference calculations are flagged with a double asterisk (**)

### Reference calculations

In the SAMPL7 host–guest challenge, we ran our reference calculations using an alchemical approach with the YANK [83] automated toolkit. With this approach we used the Hamiltonian replica-exchange sampling method [83, 84], and in some cases we could not achieve convergence in the free energy estimate even with 50 ns of simulation. For SAMPL8 we decided to take an alternative approach employing the APR method via the pAPRika toolkit. The APR method is a physical path-based method using umbrella sampling (US) and has been used in previous SAMPL challenges [18, 20]. We decided to use this approach for several reasons: (a) the use of US in APR allows for each individual and independent umbrella or state to be simulated separately. Thus, the individual umbrellas can all be simulated in parallel, allowing for fast simulations, and expedited reference calculations with modest accuracy. (b) The APR method has been used in benchmarking studies, and default setting/parameters used in the method have been established for calculating thermodynamic measurements of host–guest systems similar to those present in SAMPL8 [18, 61, 85, 86]. (c) Given that the hosts and guests in this study are relatively rigid, the binding modes are well known, we thought the use of enhanced sampling may not be necessary in the majority of cases. At the same time, perhaps using this approach would also bring insight on where enhanced sampling provides the greatest benefits.

Our in-house reference calculations were performed using the APR method [67, 87] with pAPRika 1.0.4 (https://github.com/slochower/pAPRika/tree/v1.0.4), and OpenMM 7.4.2 as the simulation engine. In total, 15 windows were used for the attach and release phases, and up to 46 umbrella sampling windows (depending on the size of the guest) during the pull phase.

The starting structures were obtained by docking using OEDock (with the Chemgauss4 scoring function) from OpenEye Toolkits (release 2019.4.2). AM1-BCC partial atomic charges were generated with oequacpac function oequacpac.OEAssignCharges(mol, oequacpac.OEAM1BCCCharges()) as implemented with OpenEye Toolkits (release 2019.4.2). Each host–guest system was solvated with 2500 TIP3P water molecules in a rectangular box whose dimensions were approximately $40 \times 40 \times 63$ cubic Å. Sodium and/or chloride counter ions (with parameters from Joung and Cheatham [88]) were added as needed to neutralize each host–guest system, and additional NaCl ions were added to obtain an ionic strength matching experimental conditions. To compare the performance of two general Force Fields (Parsley and GAFF2), the bonded and Lennard-Jones parameters for hosts and guests were assigned based on OpenFF v1.2.0 using the Open Force Field toolkit, or from GAFF2 as implemented in Antechamber [89].

The attach–pull–release windows were prepared using pAPRika 1.0.4, which consists of: adding three non-interacting anchor particles, defining host and guest anchor atoms, configuring Boresch-style restraints [90], the addition of solvent and ions, and preparation of OpenMM XML files. First, three heavy atoms for each host were defined as host anchor atoms (H1, H2, and H3), and two heavy atoms for each guest were defined as guest anchor atoms (L1, and L2). Guest anchor atom L1 was shifted to the origin, and the host–guest complex was oriented by aligning the vector formed by L1 and L2 to the *z*-axis. Three non-interacting particles, called dummy atoms (D1, D2, and D3), were added to the system along the *z*-axis below the guest molecule at distances of 6, 9, and 11.2 Å, respectively. The third dummy atom (D3) was also offset by 2.2 Å, in the *y*-axis.

As described elsewhere [67, 91], six Boresch-style restraints (one distance restraint, two angle restraints, and three torsional restraints) were used to restrain the translation and orientation of the host molecule to impose a lab frame of reference. The translation was defined by restraints on anchor atoms D1–L1, D2–D1–L1, and D3–D2–D1–L1 while the orientation was defined by D1–H1–H2, D2–D1–H1–H2, and D1–H1–H2–H3. Collectively, these six restraints were referred to as “static” restraints because they are constant throughout entire simulations. The restraint free energy of the static restraints was not included in the calculation because the restraints do not alter the internal coordinates of the host molecule and thus do not contribute to the binding free energy and serve solely to define the frame of reference.

During the attach phase, three restraints were applied to the guest molecule; two for the translation (*r* and $\theta$) and one for the orientation ($\beta$). We only restrain the polar angle of the guest orientation because the host molecules of interest are cylindrically symmetrical. The restraint free energy was obtained by scaling the force constants from 0 to 1 in 15 windows. The free energy of releasing the guest restraints in the unbound state was calculated semi-analytically, which includes the standard-state correction at 1 M. The force constants used for the host static restraints and the guest orientational restraints were: (a) distance restraints = 10.0 kcal/mol/Å^2^; (b) angle and torsional restraints = $100\,{\text {kcal}}/{\text {mol}}/{\text {rad}}^{2}$.

In the pull phase, the guest molecule was pulled from the host along a reaction coordinate defined as the distance between D1–L1. The two angles, D2–D1–L1 and D1–L1–L2, were restrained at 180$^{\circ }$ throughout the pull phase. The guest was pulled up to a distance of 18 Å  from the first window in intervals of 0.4 Å  totaling to 46 windows.

Conformational restraints were applied on the host. These are optional in APR calculations to facilitate sampling during the pulling phase [67, 85]. For CB8, eight distance “jack” restraints were used on the carbonyls to enlarge the cavity. Jack restraints with a distance of 13.5 Å  were used in previous calculations of CB7 [67] and we applied a slightly larger distance for CB8. From our initial calculations, we found that a distance of 14 Å  was enough to achieve good overlap between neighboring windows. For TEMOA, four distance jack restraints were used on the upper phenyl groups of the cavity. The same four distance jack restraints were used for TEETOA on the upper phenyl groups, with an additional two diagonal restraints on the ethyl groups to keep the groups as far apart as possible. The free energy contribution of applying conformational restraints on the host molecule was calculated in the same manner as the guest restraints and was simultaneously scaled in the attach phase (the restraint free energy was obtained by scaling the force constants using scaling coefficients from 0 to 1, thus turning on the restraints in 15 windows), as were the guest restraints. However, the free energy cost of releasing the conformational restraints in the unbound state was calculated explicitly by scaling the force constants to zero in 15 windows. The parameters used for the conformational restraints were: (a) jack distance = 14.0 Å; (b) force constant = 13.0 kcal/mol/Å^2^.

A set of flat-bottom potential restraints were placed to prevent the guest molecule from leaving the binding pocket during the attach phase. Here, we refer to these as “wall” restraints. We used them to improve convergence, especially for weak binders. We stress that these restraints do not contribute to the final, binding free energy and are only applied if the guest molecule leaves the binding site beyond a threshold in the attach phase [67]. For CB8, eight wall restraints were set on the guest relative to a carbon in each glycoluril unit. Referred to by atom name from the files provided, these were carbons C$_{2},$ C$_{6},$ C$_{10},$ C$_{14},$ C$_{18},$ C$_{22},$ C$_{26},$ and C$_{31}.$ For the TEMOA and TEETOA hosts, four wall restraints were set on the guests relative to carbons (C$_{47},$ C$_{53},$ C$_{35},$ and C$_{41}$) surrounding the center of the cavity. The parameters used for the wall restraints were: (a) wall distance = 14 Å; (b) force constant = 50 kcal/mol/Å^2^.

From a unbiased 200 ns MD simulation for the TEETOA–G1 system, TEETOAs ethyl groups were observed to be mostly in the inward orientation. However, based on the size of the guest we believed the ethyl groups would orient outwards perhaps more frequently upon complexation. To test the sensitivity of the orientation of TEETOAs ethyl groups on the predicted affinity, two separate simulations were run for this system. In the first case, the TEETOA ethyl groups were restrained toward the cavity, called “inward”, using additional dihedral restraints as “jack” host-restraints. The force constant used was 100 kcal/mol/rad${^{2}}$ and the dihedral angle was restrained at 100$^{\circ }$. In a second case, the TEETOA ethyl groups were restrained away from the cavity, called “outward”, using the same force constant but the dihedral angle restrained at $-100^{\circ }$.

All simulations were run at a constant temperature of 298.15 K using a Langevin thermostat [67, 92] with collision frequency 1.0 ${\text {ps}}^{-1}$ and the pressure is maintained at 1 atm using the Monte Carlo barostat [67, 93]. All systems were minimized up to a maximum of 5000 steps and equilibrated in the NPT ensemble for 1 ns. Production simulations (in the NPT ensemble) were run up to 30 ns per window. The non-bonded interactions were truncated with a 9.0 Å  cutoff. Long-range electrostatic interactions were handled with the particle mesh Ewald (PME) method [94, 95] while an isotropic dispersion correction [96–98] was used for the long-range van der Waals interactions. The simulation time step was set to 4 fs with Hydrogen Mass Repartitioning (HMR). Free energy quantities were estimated with thermodynamic integration (TI) and/or the Multistate Bennett Acceptance Ratio (MBAR) [99] method. The uncertainties for TI calculations were obtained using block analysis [67].

### Considering multiple protonation states of the guest

**Fig. 3:**
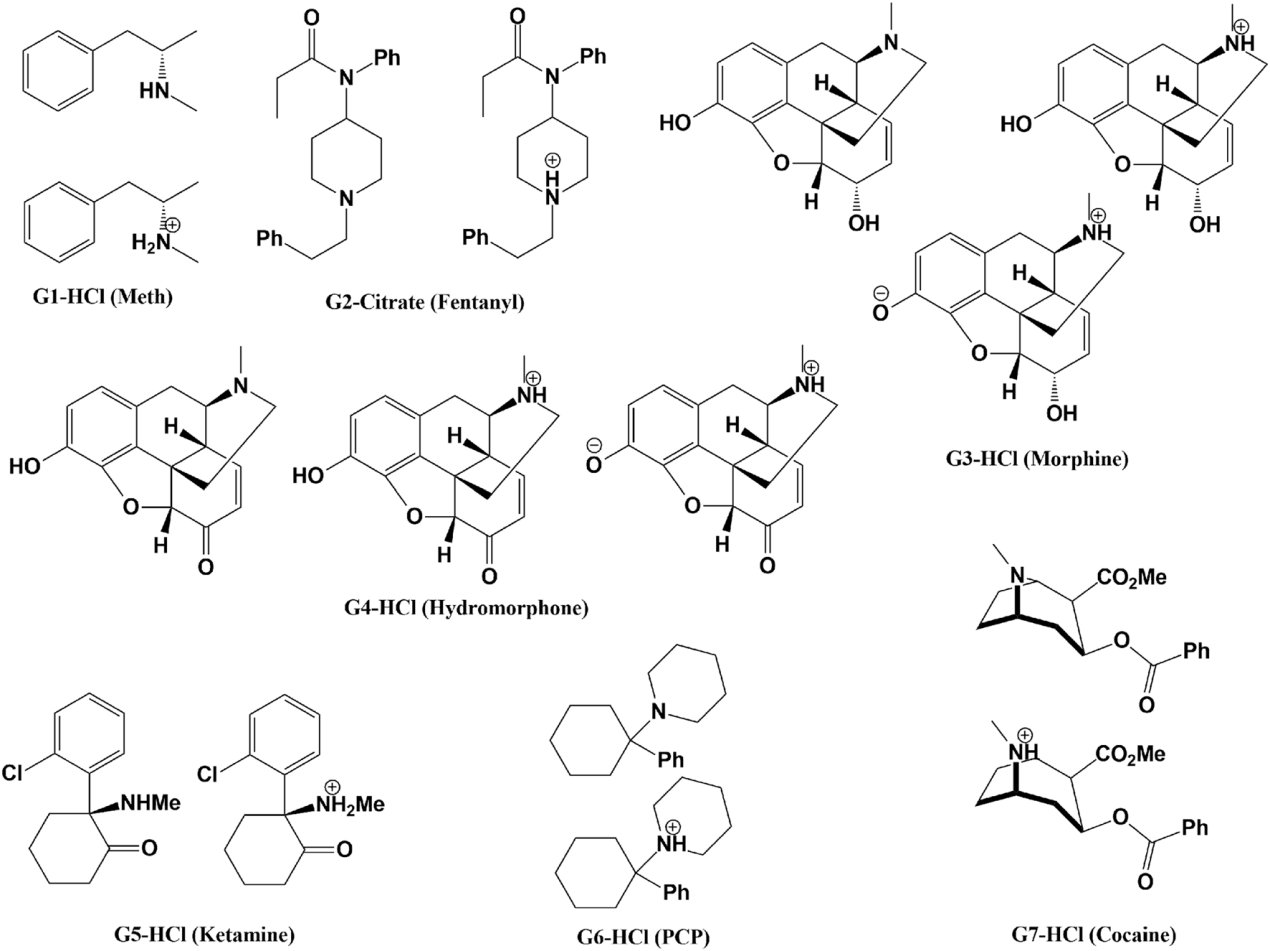
Protonation states considered for the CB8 guests. The figure shows protonation states considered at pH 7.4 for each guest in the CB8 dataset. All guests have at least two accessible protonation states, and guests G3 and G4 have three accessible states as predicted by Chemicalize from ChemAxon

CB8 guests G1 through G7 have a titratable nitrogen (Fig. 3 and Figs. S1 through S7) with predicted p*K*_a_values of 10.21, 8.77, 9.12, 9.08, 7,16, 10.56, and 8.85, respectively (determined via ChemAxon). In addition, guests G3 (morphine) and G4 (Hydromorphone) have at least one additional hydroxyl group for which the deprotonated form could possibly be relevant (Figs. S3, S4). Only three protonation states are likely populated for G3 and G4 at pH 7.4 (Fig. 3). Guest G5 has protonated (positively charged) and non-protonated (neutral) state populations of approximately 36$\%$ and 63$\%$, respectively, as determined via Chemicalize from ChemAxon. The favored neutral Ketamine state was confirmed with the OpenEye Toolkit, thus two simulations for CB8 with Ketamine (G5) were initially done. For guests G1–G4, and G6–G7, we initially did our calculations only on states with a protonated nitrogen, as these had populations of over 90$\%$ (Figs. S1 through S7).

The additional states of G1 (2 states) and G6 (2 states) are predicted to be populated near 0.15$\%$ or less, while for guests G2 (2 states), G3 (3 states), G4 (3 states), and G7 (2 states) are populated at $\approx$5$\%$ or less (Figs. S1 through S7). Thus additional states for these guests were considered only after the challenge deadline.

In the GDCC dataset, guests G1 and G2 had at least 2 accessible/populated protonation states at the experimental pH of 11.5. (Figs. S8 through S12) The guests protonation states were generated using an in-house script with OpenEye toolkits. The script also ordered the generated protonation states from the most likely state to least. The most likely state was then cross referenced with ChemAxon Chemicalize and used as the protonation state of the guest to model for free energy calculations.

### Experimental binding measurements

**Fig. 4:**
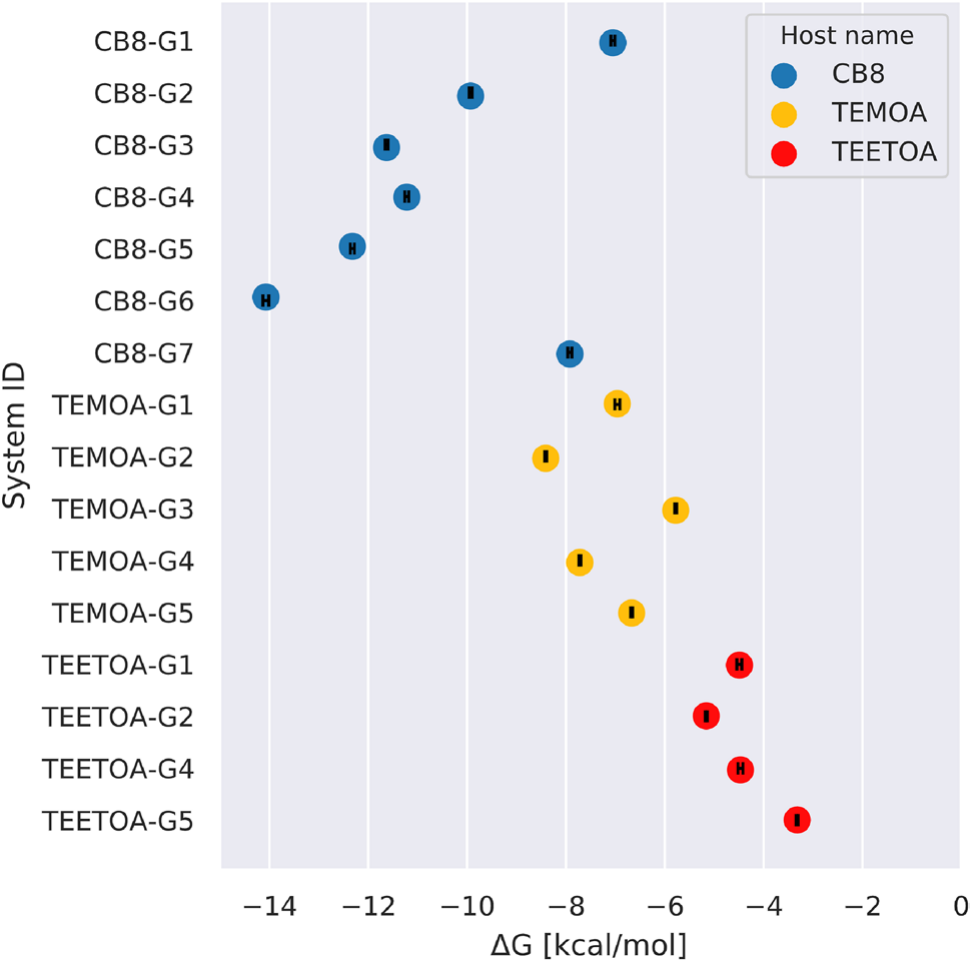
SAMPL8 host–guest experimental binding affinities. Experimental binding free energies for host–guest complexes in SAMPL8. All binding free energies ($\Delta G$) were measured via ITC and/or NMR, and are represented as colored circles [CB8 (blue), TEMOA (yellow), and TEETOA (red)]. Experimental uncertainties representing the standard error of the mean are shown as black error bars in each circle

The experimental labs of Lyle Isaacs and Bruce Gibb conducted Isothermal Titration Calorimetry (ITC) and/or Nuclear Magnetic Resonance (NMR) spectroscopy to obtain binding measurements for SAMPL8 host–guest challenge. All experimental binding data for host–guest systems are listed in Table 2, shown in Fig. 4, and in the SAMPL8 GitHub Repository (see https://github.com/samplchallenges/SAMPL8/tree/main/host_guest/Analysis/ExperimentalMeasurements). If there are any updates or changes to experimental data, the GitHub repository will provide the authoritative source.

**Table 2:** Experimental binding details for all host–guest systems

ID	Name	$K_{a}$ (M$^{-1}$)	$\Delta G$ (kcal/mol)	$\Delta H$ (kcal/mol)	$T\Delta S$ (kcal/mol)$^{\text{b}}$	*n*
CB8–G1	Methamphetamine$^{\text{a}}$ $^{\text{c}}$	1.5 × 10^5^ ± 0.1 × 10^5^	$-7.05 \pm 0.04$	$-7.8 \pm 0.3$	$-0.8 \pm 0.3$	1.00
CB8–G2	Fentanyl$^{\text{a}}$ $^{\text{c}}$	1.9 × 10^7^ ± 0.1 × 10^7^	$-9.93 \pm 0.03$	$-10.8 \pm 0.3$	$-0.9 \pm 0.3$	1.00
CB8–G3	Morphine$^{\text{a}}$ $^{\text{d}}$	3.4 × 10^8^ ± 0.2 × 10^8^	$-11.63 \pm 0.03$	$-13.6 \pm 0.4$	$-2.0 \pm 0.4$	1.00
CB8–G4	Hydromorphone$^{\text{a}}$ $^{\text{d}}$	1.7 × 10^8^ ± 0.1 × 10^8^	$-11.22 \pm 0.04$	$-15.8 \pm 0.5$	$-4.6 \pm 0.5$	1.00
CB8–G5	Ketamine$^{\text{a}}$ $^{\text{e}}$	10.9 × 10^8^ ± 0.8 × 10^8^	$-12.32 \pm 0.04$	$-17.3 \pm 0.5$	$-5.0 \pm 0.5$	1.00
CB8–G6	Phenylcyclohexylpiperidine(PCP)$^{\text{a}}$ $^{\text{e}}$	2.1 × 10^10^ ± 0.2 × 10^10^	$-14.07 \pm 0.06$	$-14.9 \pm 0.4$	$-0.8 \pm 0.5$	1.00
CB8–G7	Cocaine$^{\text{a}}$ $^{\text{c}}$	6.4 × 10^5^ ± 0.5 × 10^5^	$-7.92 \pm 0.04$	$-8.3 \pm 0.3$	$-0.3 \pm 0.3$	1.00
TEMOA–G1	3-Hydroxy-2-naphthoic acid	1.3 × 10^5^ ± 0.1 × 10^5^	$-6.96 \pm 0.05$	$-17.0 \pm 1.0$	$10.0 \pm 1.0$	1.00
TEMOA–G2	*p*-Bromophenol	14.8 × 10^5^ ± 0.6 × 10^5^	$-8.41 \pm 0.02$	$-15.7 \pm 0.2$	$7.2 \pm 0.2$	1.00
TEMOA–G3	Cyclopentylacetic acid	17.4 × 10^3^ ± 0.7 × 10^3^	$-5.78 \pm 0.02$	$-7.9 \pm 0.2$	$2.2 \pm 0.2$	1.00
TEMOA–G4	Piperonylic acid	4.6 × 10^5^ ± 0.2 × 10^5^	$-7.72 \pm 0.02$	$-17.7 \pm 0.3$	$10.0 \pm 0.3$	1.00
TEMOA–G5	*p*-Toluic acid	7.8 × 10^4^ ± 0.3 × 10^4^	$-6.67 \pm 0.02$	$-14.2 \pm 0.8$	$7.6 \pm 0.7$	1.00
TEETOA–G1	3-Hydroxy-2-napththoic acid	2.0 × 10^3^ ± 0.2 × 10^3^	$-4.49 \pm 0.05$	$-13.6 \pm 0.2$	$9.2 \pm 0.1$	1.00
TEETOA–G2	*p*-Bromophenol	6.1 × 10^3^ ± 0.2 × 10^3^	$-5.16 \pm 0.02$	$-11.6 \pm 0.3$	$6.5 \pm 0.3$	1.00
TEETOA–G3	Cyclopentylacetic acid$^{\text{f}}$	ND ± ND	ND ± ND	ND ± ND	ND ± ND	1.00
TEETOA–G4	Piperonylic acid	1.9 × 10^3^ ± 0.2 × 10^3^	$-4.47 \pm 0.05$	$-13.0 \pm 0.9$	$8.5 \pm 0.8$	1.00
TEETOA–G5	*p*-Toluic acid$^{\text{g}}$	2.7 × 10^2^ ± 0.1 × 10^2^	$-3.32 \pm 0.02$	ND ± ND	ND ± ND	1.00

All quantities are reported as point estimate ± statistical error from the ITC data fitting procedure. The upper bound ($1\%$) was used for errors reported to be $<1\%$. For the CB8 dataset, concentration error had not been factored in to the original error estimates, so we included a 3% relative uncertainty in the titrant concentration and we assumed the stoichiometry coefficient to be fitted to the ITC data [20]. For the TEMOA/TEETOA sets, provided uncertainties already include concentration error. $\Delta G$ was obtained from $K_{a}$ via the standard thermodynamic equation. The average $\Delta H$ and $\Delta G$ values were then used to calculate an average $-T\Delta S$, and the corresponding standard deviations calculated using the standard equation for the propagation of uncertainties for subtraction. The deviations in log$K_{a}$ and $\Delta G$ were obtained by using the standard equation for the propagation of uncertainties for logarithms. Binding measurements not detected or not measured are labeled with **ND**.

$^{\text{a}}$Statistical errors were propagated from the $K_{a}$ measurements

$^{\text{b}}$All experiments were performed at 298 K

$^{\text{c}}$Direct ITC titration

$^{\text{d}}$Competitive ITC titration with C1

$^{\text{e}}$Competitive ITC titration with C2

$^{\text{f}}$Binding is too weak to be observed by $^{1}$H NMR or ITC

$^{\text{g}}$Determined by $^{1}$H NMR spectroscopy

Briefly, ITC and/or NMR experiments were performed at 298 K. The CB8–G2 host–guest 1:2 binding value is also available in Table 2. Experimental binding measurements for CB8 were done in 20 mM sodium phosphate buffer at pH 7.4. Guest concentrations were in the 0.5 mM to 1.5 mM range and CB8 concentrations are 0.025 mM to 0.1 mM. All CB8 binding stoichiometries were validated by repeated ITC experiments and by NMR spectroscopy binding studies. For more details, please refer to the associated experimental paper [57].

Binding constants for the GDCC dataset were measured in 10 mM sodium phosphate buffer at pH $11.5 \pm 0.1.$ Binding measurements were done by ITC or NMR. In general, binding determination was carried out in triplicate using ITC, and the affinity constants ($K_{a}$) and binding enthalpies ($\Delta H$) were extracted and used to derive $\Delta G$ and $-T \Delta S$. NMR was used for very weak binders and $\Delta G$ was extracted from $K_{a}$. Binding of one guest (G3) to TEETOA was undetectable by ITC and NMR. To eliminate any neutralization contributions to $\Delta H$, attempts were made to ensure all solution concentrations for each experiment were within $\pm 0.05$ of tolerance. For each experiment, fresh solutions of host and guest were used to gather the data. In addition, waters of hydration are determined by qNMR using sodium ethanesulfonate as the water soluble internal standard of a known precise concentration. This standard will ensure the highest accuracy of host solution concentration and avoid misfits from concentration errors. For more details on GDCC experimental measurements, readers are advised to refer to the experimental literature.

## Results and discussion

For the SAMPL8 host–guest challenge, we find that binding free energy predictions are more accurate for GDCC hosts compared to CB8. This was also the case in previous SAMPL iterations despite CB8 and TEMOA being featured in those iterations. Then, we discuss the results for ranked methods and identify the top performing methods, compare ranked methods with each other, and discuss success and/or failures of methods. Separately, we also compare all methods (including non-ranked submissions and reference calculations) to probe the sensitivity of results to changes in protocol and/or method.

First, we break down participation and submission statistics for SAMPL8. We received a total of 51 submissions from 11 different groups, with 34 for CB8 and 17 for GDCC. The challenge involved 18 ranked submissions total, 10 of which were for CB8 and 8 for GDCC, and which constitute our primary focus in analysis (see Sect. 3.1). Five groups submitted predictions for both the CB8 and GDCC datasets, providing the opportunity to compare the reliability of these approaches across multiple hosts.

Only two groups in total submitted binding enthalpy predictions, and both were for CB8. One of the approaches had excellent predictive accuracy and correlation with experimental binding enthalpies (see Fig. S13). However, both methods performed particularly poorly at predicting binding free energies.

Most of the participants generated the initial host–guest complex for their calculations by docking, using various docking software. Methods also differed in how they modeled the buffer conditions, with some using an experimental ionic strength with sodium and chloride ions, while others using only neutralizing counter ions.

### Ranked submissions

**Fig. 5:**
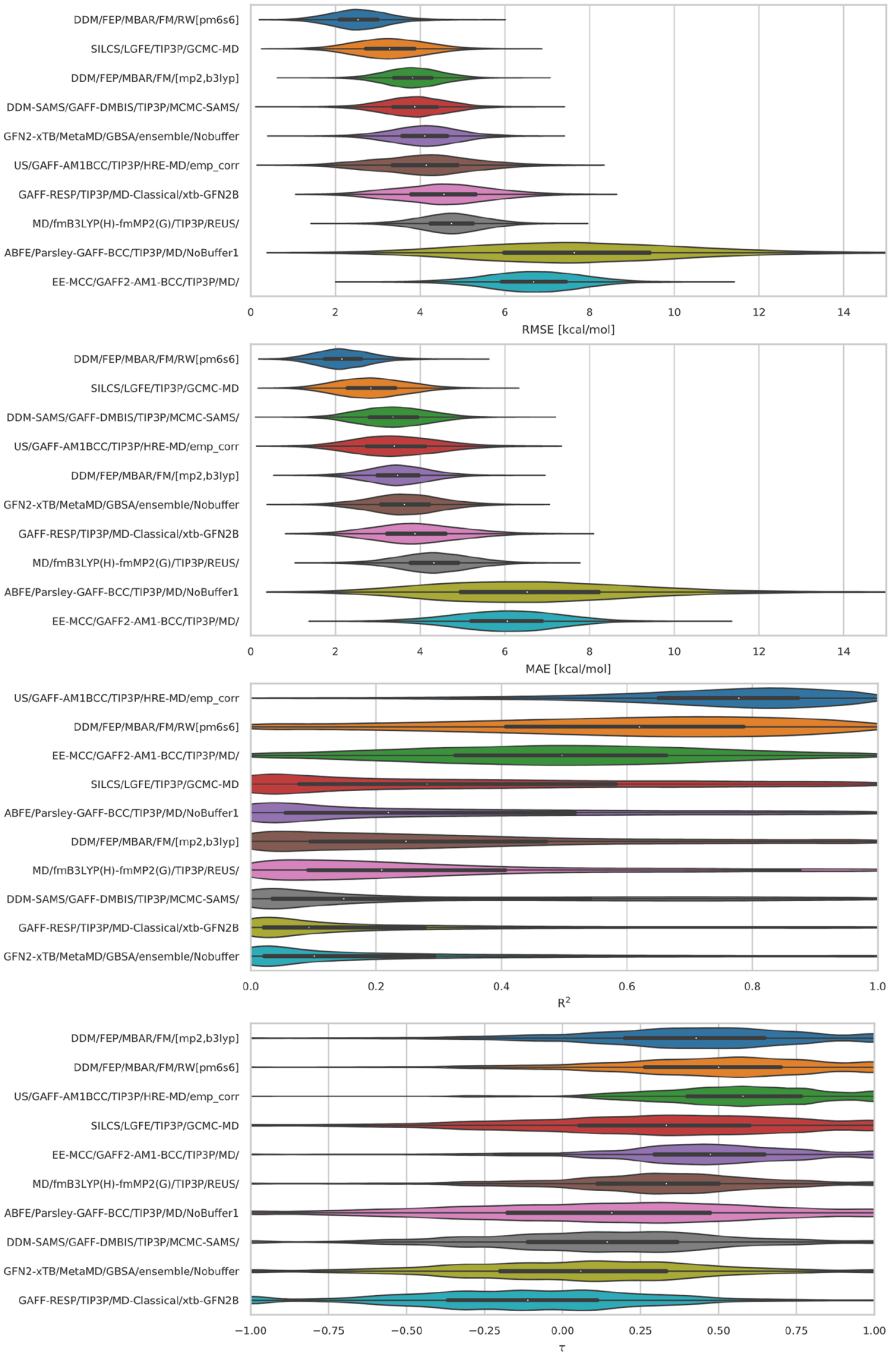
CB8 error and correlation metrics for ranked methods. Shown here are violin plots of the distribution of performance for CB8. The error and correlation metrics (from top to bottom) include RMSE, MAE, R$^{2}$, and $\tau$. The plots describe the shape of the distribution for each prediction in the dataset. For each error metric the median is indicated by a white circle in the violin plot. The black horizontal bars represent the first and third quartiles. The metrics and the relevant plots were generated by bootstrapping samples with replacement (including experimental uncertainties)

Performance statistics for most ranked methods in CB8 and GDCC were relatively similar. The similarity in the results is likely due to these methods using the same energy model (GAFF and TIP3P). There were a few methods with slight differences in the chosen energy model (such as using GAFF and TIP4P rather than GAFF and TIP3P) or use QM/DFT based approaches, yet have similar results to those with GAFF/TIP3P. However, a couple methods stand out as clear top performers or perform particularly poorly (Figs. 5, 7).

#### CB8: “Drugs of Abuse Challenge”

In the CB8 drugs of abuse challenge, the top performing method was a force matching (FM) method, *DDM/FEP/MBAR/FM/RW[pm6s6]*, with RMSE, MAE, and R$^{2}$ values of 2.43 kcal/mol, 2.03 kcal/mol, and 0.59, respectively. The error and correlation metrics for the FM method were in general the best, while in few exceptions it was at least top 2. Behind the FM method was the *SILCS/LGFE/TIP3P/GCMC-MD* method with RMSE, MAE, and R$^{2}$ values of 3.06 kcal/mol, 2.59 kcal/mol, and 0.40 respectively. Both *DDM/FEP/MBAR/FM/RW[pm6s6]* and *SILCS/LGFE/TIP3P/GCMC-MD* methods achieved the best error metrics, however, they did not have the best correlation, which will be discussed shortly. Ordered by the RMSE error metric, the next six methods had values ranging from 3.77 kcal/mol to 4.68 kcal/mol while the lowest two methods ranged from 5.72 to 6.64 kcal/mol (Table 3; Fig. 5). The results showcase the diverse and variable performance of methods for this challenge.

**Table 3:** Error metrics for all (ranked and non-ranked) SAMPL8 methods for all host–guest systems

ID	Sid	RMSE (kcal/mol)	MAE (kcal/mol)	ME (kcal/mol)	R$^{2}$	m	$\tau$
CB8							
*SILCS/LGFE/TIP3P/GCMC-MD/rew*	5	1.96 [1.09, 4.61]	1.71 [0.87, 4.09]	− 0.23 [− 2.76, 2.29]	0.38 [0.00, 0.96]	0.58 [− 0.71, 1.80]	0.52 [− 0.58, 1.00]
DDM/FEP/MBAR/FM/RW[pm6s6]*	28	2.46 [1.26, 3.94]	2.03 [1.03, 3.50]	0.68 [− 1.37, 2.71]	0.59 [0.03, 0.98]	1.22 [− 0.05, 2.32]	0.52 [− 0.33, 1.00]
*ML/NNET/CORINA-descriptors2*	10	2.73 [1.58, 4.18]	2.41 [1.29, 3.85]	− 1.43 [− 3.42, 0.66]	0.01 [0.00, 0.95]	0.04 [− 0.71, 0.63]	0.14 [− 1.00, 1.00]
SILCS/LGFE/TIP3P/GCMC-MD*	3	3.06 [1.65, 5.07]	2.59 [1.31, 4.64]	− 2.46 [− 4.50, − 0.44]	0.40 [0.00, 0.96]	0.29 [− 0.49, 1.19]	0.43 [− 0.65, 1.00]
*SILCS/LGFE/TIP3P/GCMC-MD/G5*	4	3.16 [1.69, 5.19]	2.67 [1.35, 4.73]	− 2.53 [− 4.61, − 0.46]	0.33 [0.00, 0.96]	0.27 [− 0.54, 1.12]	0.43 [− 0.71, 1.00]
*DDM/FEP/MBAR/Paramchem/FM/[gfn2gfn2]*	19	3.47 [2.19, 4.94]	3.23 [1.89, 4.67]	− 1.48 [− 3.93, 1.12]	0.43 [0.00, 0.97]	− 0.32 [− 0.94, 0.39]	− 0.33 [− 1.00, 0.56]
*ML/NNET/CORINA-descriptors1*	9	3.50 [2.11, 4.96]	3.02 [1.58, 4.66]	− 2.78 [− 4.59, − 0.87]	0.14 [0.00, 0.95]	0.15 [− 0.47, 0.71]	0.33 [− 0.71, 1.00]
*DDM/FEP/MBAR/FM/[pm6s6]*	22	3.60 [2.36, 4.99]	3.23 [1.91, 4.74]	3.23 [1.66, 4.73]	0.64 [0.04, 0.99]	0.92 [0.08, 1.69]	0.62 [− 0.20, 1.00]
*DDM/FEP/MBAR/FM/RW/[wb97xd,s6]*	29	3.67 [2.37, 5.12]	3.29 [1.93, 4.85]	2.89 [0.83, 4.79]	0.38 [0.00, 0.98]	0.75 [− 0.29, 1.83]	0.33 [− 0.53, 1.00]
*DDM/FEP/MBAR/Paramchem/FM/[C36S6]*	18	3.73 [2.21, 5.28]	3.17 [1.65, 4.94]	3.08 [1.17, 4.89]	0.41 [0.01, 0.96]	0.74 [− 0.31, 1.82]	0.33 [− 0.33, 1.00]
DDM/FEP/MBAR/FM/[mp2,b3lyp]*	15	3.77 [2.48, 5.26]	3.39 [2.05, 4.94]	2.50 [0.09, 4.66]	0.20 [0.00, 0.96]	0.57 [− 0.34, 2.40]	0.52 [− 0.33, 1.00]
DDM-SAMS/GAFF-DMBIS/TIP3P/MCMC-SAMS/*	2	3.82 [2.33, 5.43]	3.25 [1.77, 5.08]	1.74 [− 1.08, 4.29]	0.11 [0.00, 0.95]	0.49 [− 1.29, 2.47]	0.05 [− 0.67, 0.88]
APR/GAFF2-AM1BCC/TIP3P/US/TI**	33	3.85 [2.35, 5.46]	3.38 [1.90, 5.15]	2.87 [0.65, 5.01]	0.53 [0.08, 0.97]	1.18 [0.29, 2.75]	0.52 [− 0.20, 1.00]
APR/GAFF2-AM1BCC/TIP3P/US/MBAR**	34	3.89 [2.32, 5.60]	3.41 [1.89, 5.19]	2.86 [0.59, 5.03]	0.63 [0.21, 0.96]	1.40 [0.54, 2.91]	0.52 [− 0.20, 1.00]
*DDM/FEP/MBAR/Paramchem/FM/RW/[wb97xd]*	26	3.90 [2.08, 5.96]	3.41 [1.81, 5.33]	0.19 [− 3.01, 3.07]	0.53 [0.05, 0.97]	1.68 [0.12, 3.21]	0.62 [− 0.18, 1.00]
GFN2-xTB/MetaMD/GBSA/ensemble/Nobuffer*	1	4.06 [2.54, 5.68]	3.60 [2.00, 5.31]	2.89 [0.46, 5.15]	0.01 [0.00, 0.92]	0.08 [− 0.89, 1.12]	− 0.05 [− 0.87, 0.79]
APR/OPENFF1.2.0-AM1BCC/TIP3P/US/TI**	31	4.07 [2.20, 5.89]	3.18 [1.53, 5.38]	2.89 [0.53, 5.22]	0.11 [0.00, 0.89]	0.38 [− 0.50, 1.58]	0.24 [− 0.58, 0.88]
APR/OPENFF1.2.0-AM1BCC/TIP3P/US/MBAR**	32	4.14 [2.47, 5.88]	3.45 [1.81, 5.47]	3.21 [0.97, 5.40]	0.14 [0.00, 0.92]	0.40 [− 0.48, 1.55]	0.24 [− 0.65, 0.88]
US/GAFF-AM1BCC/TIP3P/HRE-MD/emp_corr*	11	4.15 [1.96, 6.42]	3.37 [1.60, 5.65]	2.20 [− 0.70, 5.08]	0.74 [0.28, 0.99]	2.00 [0.86, 3.85]	0.43 [− 0.20, 1.00]
*LiGaMD/q4MD/TIP4P/enhanced-sampling*	16	4.28 [2.43, 6.39]	3.60 [1.90, 5.80]	− 2.35 [− 5.22, 0.57]	0.03 [0.00, 0.95]	− 0.17 [− 1.82, 0.97]	− 0.24 [− 1.00, 0.76]
*DDM/FEP/MBAR/C36*	17	4.44 [2.51, 6.23]	3.76 [1.91, 5.78]	3.58 [1.33, 5.74]	0.24 [0.00, 0.93]	0.60 [− 0.42, 1.85]	0.24 [− 0.41, 1.00]
*GAFF-RESP/TIP3P/MD/xtb-GFN2B/Boltz-Avg*	8	4.55 [2.64, 6.42]	3.98 [2.15, 5.93]	− 0.92 [− 4.14, 2.81]	0.00 [0.00, 0.95]	0.04 [− 1.70, 1.68]	0.14 [− 1.00, 1.00]
GAFF-RESP/TIP3P/MD-Classical/xtb-GFN2B*	7	4.60 [2.50, 6.87]	3.87 [2.08, 6.13]	1.50 [− 1.90, 4.90]	0.01 [0.00, 0.94]	− 0.18 [− 1.62, 1.48]	− 0.24 [− 1.00, 0.60]
*DDM/FEP/MBAR/FM/[mp2s6]*	21	4.68 [3.08, 6.22]	4.09 [2.36, 5.96]	4.09 [2.00, 5.93]	0.37 [0.00, 0.96]	0.74 [− 0.30, 2.02]	0.43 [− 0.47, 1.00]
MD/fmB3LYP(H)-fmMP2(G)/TIP3P/REUS/*	13	4.68 [3.19, 6.20]	4.27 [2.64, 5.95]	2.52 [− 0.86, 5.26]	0.16 [0.00, 0.94]	0.74 [− 0.37, 3.25]	0.33 [− 0.37, 1.00]
*DDM/FEP/MBAR/FM/[gfn2,s6]*	23	5.13 [3.64, 6.68]	4.79 [3.09, 6.47]	4.50 [2.23, 6.45]	0.23 [0.00, 0.98]	0.52 [− 0.72, 1.52]	0.43 [− 0.60, 1.00]
*DDM/FEP/MBAR/FM/RW/[blyp,s6]*	24	5.13 [2.52, 8.00]	4.28 [2.32, 6.87]	0.08 [− 4.29, 3.49]	0.42 [0.01, 0.98]	1.80 [− 0.14, 4.06]	0.52 [− 0.26, 1.00]
*DDM/FEP/MBAR/FM/[pm6pm6]*	20	5.29 [2.87, 7.44]	4.22 [1.99, 6.80]	− 3.94 [− 6.68, − 1.10]	0.13 [0.00, 0.94]	− 0.31 [− 1.63, 0.35]	− 0.52 [− 1.00, 0.53]
*DDM/FEP/MBAR/FM/RW/[blyp,s6BLUR]*	25	5.53 [3.44, 7.69]	5.01 [3.11, 7.11]	3.90 [0.65, 6.83]	0.56 [0.06, 0.95]	1.73 [0.34, 3.63]	0.52 [− 0.29, 1.00]
*ABFE/Parsley-GAFF-BCC/TIP3P/MD/NoBuffer2*	14	5.72 [3.24, 13.27]	5.16 [2.57, 11.84]	5.16 [− 1.01, 11.36]	0.22 [0.00, 0.95]	0.51 [− 2.41, 3.74]	0.33 [− 0.79, 1.00]
ABFE/Parsley-GAFF-BCC/TIP3P/MD/NoBuffer1*	30	5.72 [3.22, 13.09]	5.16 [2.53, 11.73]	5.16 [− 1.01, 11.30]	0.22 [0.00, 0.95]	0.51 [− 2.41, 3.72]	0.33 [− 0.79, 1.00]
*DDM/FEP/MBAR/FM/RW/[wb97xdBLUR]*	27	5.98 [4.03, 7.91]	5.54 [3.65, 7.52]	4.40 [0.97, 7.30]	0.62 [0.06, 0.97]	1.92 [0.34, 3.70]	0.43 [− 0.29, 1.00]
EE-MCC/GAFF2-AM1-BCC/TIP3P/MD/*	6	6.64 [4.39, 8.82]	5.97 [3.63, 8.42]	5.97 [3.39, 8.42]	0.48 [0.04, 0.95]	1.21 [0.11, 2.65]	0.39 [− 0.29, 1.00]
*US/GAFF-AM1BCC/TIP3P/HRE-MD*	12	9.36 [5.05, 18.19]	8.80 [4.07, 16.52]	8.80 [1.24, 16.37]	0.70 [0.00, 0.97]	1.77 [− 1.76, 5.59]	0.52 [− 0.60, 1.00]
GDCC–TEMOA and TEETOA							
DDM/AMOEBA/BAR*	44	0.88 [0.46, 1.56]	0.72 [0.36, 1.36]	0.10 [− 0.72, 0.84]	0.78 [0.20, 0.98]	0.97 [0.43, 1.38]	0.79 [0.24, 1.00]
ATM/GAFF2-AM1BCC/TIP3P/HREM*	37	1.59 [1.10, 3.96]	1.25 [0.87, 3.43]	− 0.39 [− 2.33, 1.83]	0.88 [0.14, 0.98]	1.67 [0.46, 3.03]	0.71 [0.00, 1.00]
PMF/GAFF2-AM1BCC/TIP3P/MD-US*	38	1.59 [1.13, 4.02]	1.31 [0.88, 3.53]	− 0.16 [2.17, 2.03]	0.79 [0.06, 0.97]	1.51 [0.30, 2.97]	0.71 [− 0.08, 1.00]
*AM1BCC/MMPBSA/TIP4PEW/MD_NR3*	42	1.98 [1.15, 3.28]	1.69 [0.91, 2.96]	0.77 [− 0.82, 2.35]	0.00 [0.00, 0.83]	0.02 [− 0.75, 0.70]	0.18 [− 0.76, 0.82]
AM1BCC/MMPBSA/TIP4PEW/MD*	43	2.05 [1.20, 3.30]	1.65 [0.91, 2.97]	1.05 [− 0.52, 2.57]	0.02 [0.00, 0.85]	0.06 [− 0.66, 0.75]	0.18 [− 0.71, 0.83]
*AM1BCC/MMPBSA/TIP4PEW/MD_NR2*	41	2.10 [1.20, 3.43]	1.69 [0.93, 3.10]	1.04 [− 0.57, 2.67]	0.00 [0.00, 0.84]	0.02 [− 0.75, 0.69]	0.18 [− 0.77, 0.82]
ML/NNET/CORINA-descriptors-8*	39	2.39 [1.44, 3.85]	2.16 [1.16, 3.51]	0.58 [− 1.44, 2.58]	0.60 [0.00, 0.94]	− 0.35 [− 1.13, 0.38]	− 0.64 [− 1.00, 0.57]
SILCS/LGFE/TIP3P/GCMC-MD*	36	2.40 [1.38, 3.75]	2.10 [1.12, 3.38]	− 0.24 [− 2.17, 1.70]	0.26 [0.00, 0.89]	− 0.32 [− 1.05, 0.47]	− 0.29 [− 1.00, 0.57]
*SILCS/LGFE/TIP3P/GCMC-MD_NR*	35	2.51 [1.34, 4.50]	1.81 [1.02, 3.90]	− 1.69 [− 3.66, 0.26]	0.00 [0.00, 0.88]	− 0.01 [− 1.07, 0.92]	0.07 [− 0.83, 0.83]
APR/OPENFF1.2.0-AM1BCC/TIP3P/US/TI**	48	2.97 [1.27, 4.80]	2.24 [0.99, 4.07]	− 0.76 [− 3.09, 1.36]	0.48 [0.09, 0.94]	1.55 [0.43, 3.44]	0.50 [− 0.09, 0.92]
APR/OPENFF1.2.0-AM1BCC/TIP3P/US/MBAR**	49	2.98 [1.31, 4.86]	2.24 [1.02, 4.07]	− 0.83 [− 3.14, 1.24]	0.48 [0.09, 0.92]	1.54 [0.39, 3.48]	0.50 [− 0.08, 0.92]
APR/GAFF2-AM1BCC/TIP3P/US/MBAR**	51	3.24 [1.27, 5.29]	2.30 [0.97, 4.37]	− 1.49 [− 3.82, 0.62]	0.35 [0.02, 0.90]	1.24 [0.07, 3.24]	0.43 [− 0.28, 0.84]
APR/GAFF2-AM1BCC/TIP3P/US/TI**	50	3.31 [1.41, 5.30]	2.47 [1.11, 4.45]	− 1.29 [− 3.68, 0.96]	0.26 [0.00, 0.87]	1.08 [− 0.11, 3.24]	0.29 [− 0.36, 0.83]
*LiGaMD/GAFF2/RESP/TIP4P/Sampling*	47	4.48 [1.46, 6.77]	3.07 [1.10, 5.66]	1.72 [− 1.10, 4.79]	0.00 [0.00, 0.76]	− 0.02 [− 1.78, 1.92]	0.07 [− 0.67, 0.75]
DDM/C36/TIP3P/MD/MBAR*	45	4.52 [2.01, 6.71]	3.45 [1.62, 5.84]	− 3.45 [− 5.79, − 1.34]	0.04 [0.00, 0.78]	0.35 [− 0.98, 2.00]	0.29 [− 0.57, 0.92]
MD/ParamChem/TIP3P/REUS/*	46	4.91 [2.50, 7.18]	3.95 [2.02, 6.33]	− 3.90 [− 6.29, − 1.69]	0.01 [0.00, 0.68]	0.18 [− 1.18, 1.63]	0.04 [− 0.58, 0.76]
*AM1BCC/MMPBSA/TIP4PEW/MD_NR1*	40	9.26 [7.30, 11.28]	8.93 [7.00, 10.95]	8.93 [7.00, 10.95]	0.00 [0.00, 0.73]	0.06 [− 1.18, 0.98]	0.18 [− 0.67, 0.74]

The root mean square error (RMSE), mean absolute error (MAE), signed mean error (ME), coefficient of correlation (R$^{2}$), slope (m), and Kendall’s rank correlation coefficient (Tau) were computed via bootstrapping with replacement. Shown are results for individual host categories, with upper and lower bounds of 95% confidence intervals shown in brackets. Statistics do not include optional host–guest systems CB8–G8, CB8–G9, and TEMOA–G3. Each method has an assigned unique submission ID (sid). An asterisk next to the method’s name denotes a ranked submission, and a double asterisk denotes a reference calculation

Different metrics can be used to rank methods, and the ranking can be different depending on the metric chosen. For example, the MAE can be used to rank the submissions for SAMPL8, since this error metric is not as sensitive (compared to RMSE or R$^{2}$) to outliers and it directly measures a method’s accuracy. Using MAE we see a similar ranking of methods as with RMSE (Fig. 5).

**Fig. 6:**
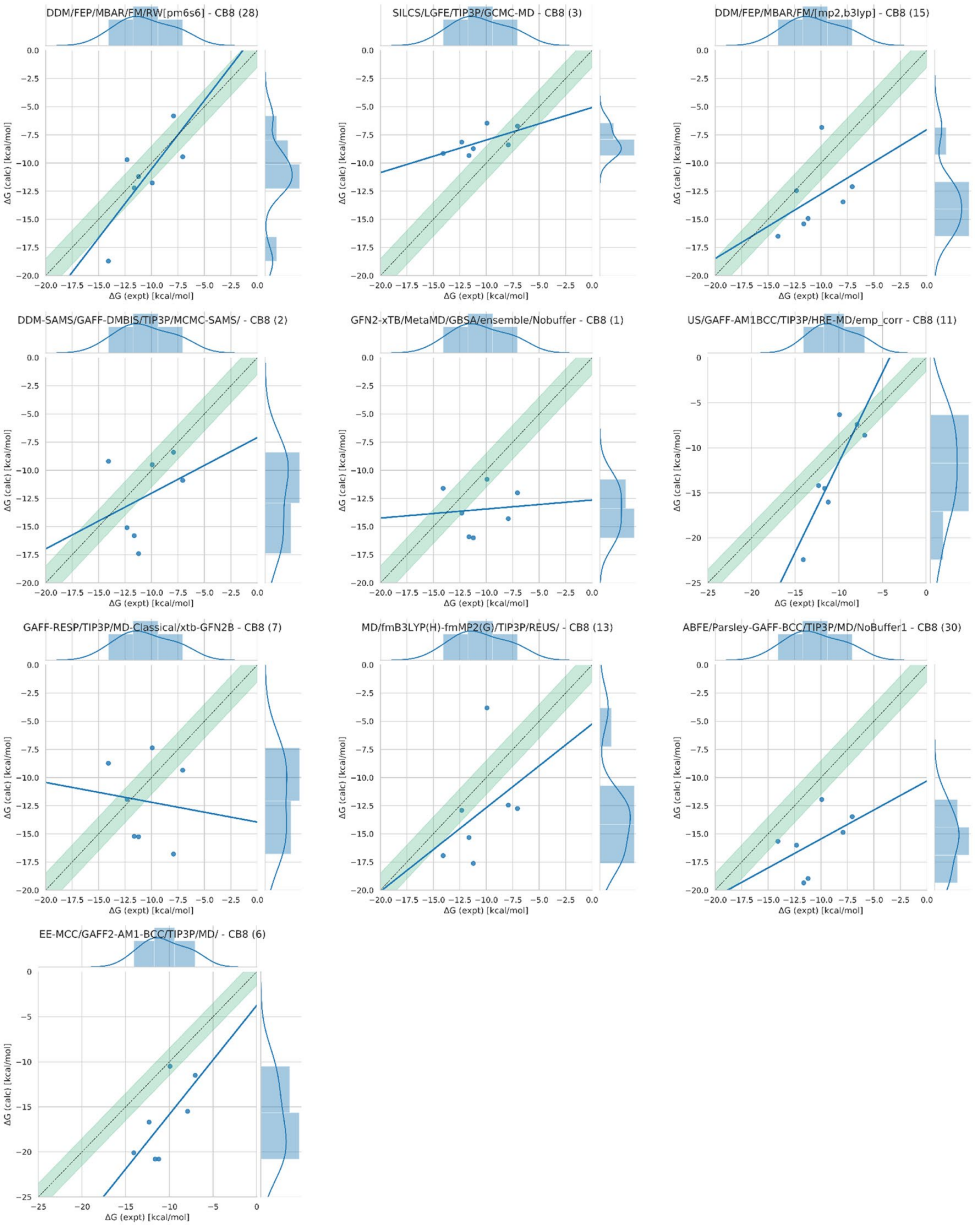
CB8 correlation plots for ranked methods. Shown here are correlation plots comparing calculated versus experimental values for the *DDM/FEP/MBAR/FM/RW[pm6s6]*, *SILCS/LGFE/TIP3P/GCMC-MD*, *DDM/FEP/MBAR/FM/[mp2,b3lyp]*, *DDM-SAMS/GAFF-DMBIS/TIP3P/MCMC-SAMS/*, *GFN2-xTB/MetaMD/GBSA/ensemble/Nobuffer*, *US/GAFF-AM1BCC/TIP3P/HRE-MD/emp_corr*, *GAFF-RESP/TIP3P/MD-Classical/xtb-GFN2B*, *MD/fmB3LYP(H)-fmMP2(G)/TIP3P/REUS/*, *ABFE/Parsley-GAFF-BCC/TIP3P/MD/NoBuffer1*, and *EE-MCC/GAFF2-AM1-BCC/TIP3P/MD* ranked predictions for the CB8 dataset

The correlation metrics for all methods were relatively poor (Fig. 6) for this dataset. Only two methods achieved an R$^{2}$ or a $\tau$ value over 0.50 (Table 3). The statistics suggest there may be some systematic error for a few methods. For example, some methods achieve high correlations along with low accuracy, indicating systematic errors (i.e. the *US/GAFF-AM1BCC/TIP3P/HRE-MD/emp_corr* method had high correlation with R$^{2}$ of 0.74, but RMSE and MAE values that were poor at 4.15 and 3.37 kcal/mol, respectively).

It is worth noting that two sets of binding enthalpy predictions were submitted for CB8, but this was too few to allow statistical analysis. The methods were the *ABFE/Parsley-GAFF-BCC/TIP3P/MD/NoBuffer1* and the more accurate (for predicting binding enthalpy) entropy-enthalpy based method *EE-MCC/GAFF2-AM1-BCC/TIP3P/MD/*. Although the *EE-MCC/GAFF2-AM1BCC/TIP3P/MD* method yielded poor binding free energy predictions, binding enthalpy predictions were within 2 kcal/mol for 4/7 systems. On the other hand, the *ABFE/Parsley-GAFF-BCC/TIP3P/MD/NoBuffer1* predicts 1/7 systems within 2 kcal/mol. The correlation was modest with a few outliers for the entropy-enthalpy method, while there was a larger error and there appeared to be some systematic errors for the *ABFE/Parsley-GAFF-BCC/TIP3P/MD/NoBuffer1* method (Fig. S13). The *EE-MCC/GAFF2-AM1-BCC/TIP3P/MD/* and *ABFE/Parsley-GAFF-BCC/TIP3P/MD/NoBuffer1* methods used the same energy model, but the *ABFE/Parsley-GAFF-BCC/TIP3P/MD/NoBuffer1* method did not model the buffer concentration. These modeling differences indeed seem to affect results substantially, with the RMSE differing by about 1 kcal/mol. The effect of buffer conditions on the binding enthalpy appears to be more significant for specific systems, and these observations warrant further studies.

#### GDCCs: sterics and flexibility challenge

**Fig. 7:**
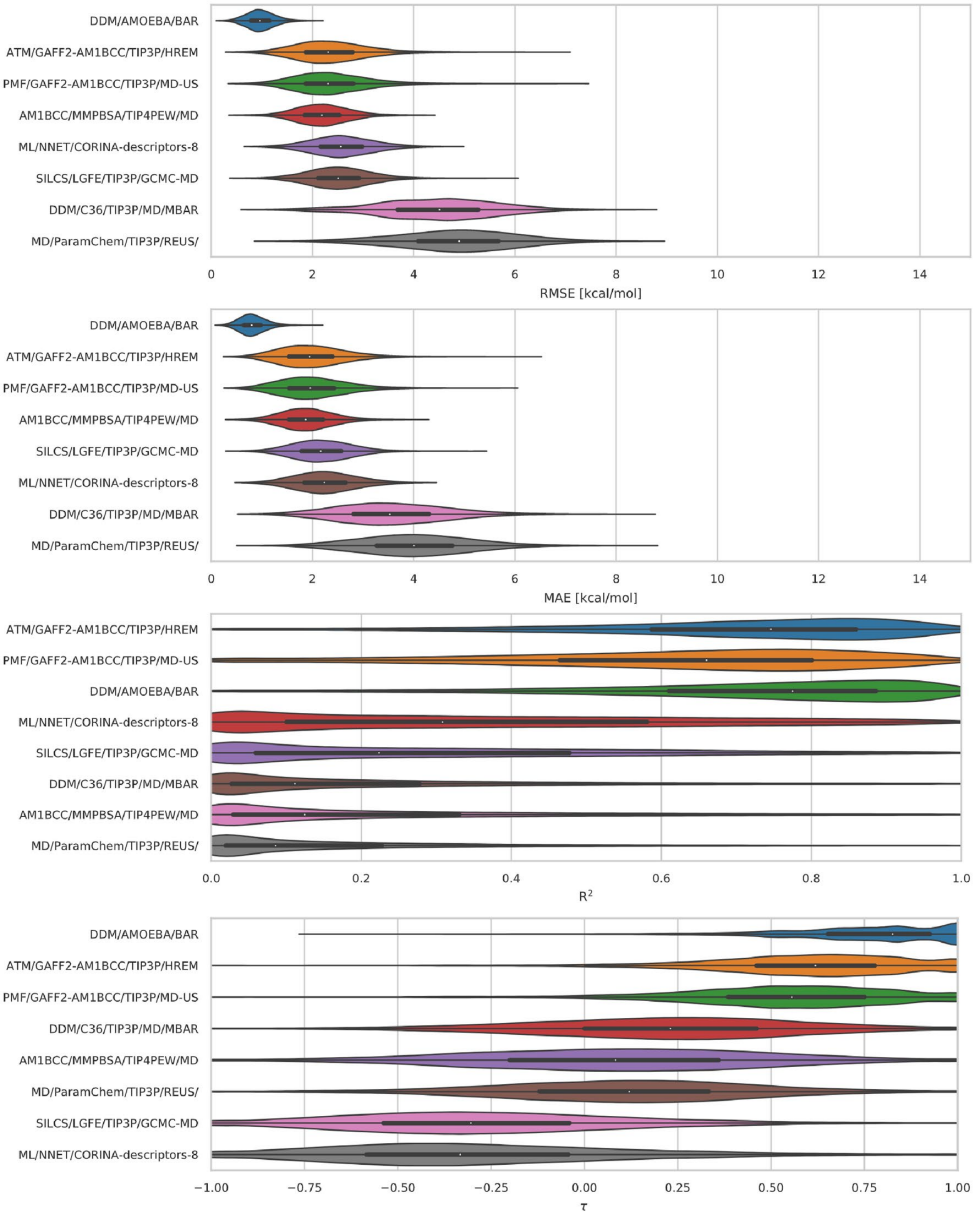
GDCC (TEMOA and TEETOA) error and correlation metrics for ranked methods. Shown here are violin plots of the distribution of performance for GDCC hosts. The error and correlation metrics (from top to bottom) include RMSE, MAE, R$^{2}$, and $\tau$. The plots describe the shape of the distribution for each prediction in the dataset. For each error metric the median is indicated by a white circle in the violin plot. The black horizontal bars represent the first and third quartiles. The metrics and the relevant plots were generated by bootstrapping samples with replacement (including experimental uncertainties)

Overall the predictive accuracy of methods for the GDCC dataset was relatively good. Several methods achieved RMSE and MAE values below 2 kcal/mol, while the majority were below 3 kcal/mol. Half of the methods had R$^{2}$ and $\tau$ values over 0.5. Overall, the top performing method for the GDCC dataset was *DDM/AMOEBA/BAR*, which had the best RMSE and MAE values of 0.88 and 0.72 kcal/mol, as well as $\tau$ values of 0.79 (Table 3; Fig. 7). The *ATM/GAFF2-AM1BCC/TIP3P/HREM* method came in second overall for the GDCC dataset. Although we observe that computational predictive power is higher overall for the GDCC dataset, there are still methods which have very poor predictive power with RMSE and MAE values as high as 4.91 and 3.95 kcal/mol and coefficients of determination as low as 0.01 (Fig. 7; Table 3).

#### Binding free energy of some host–guest systems were more difficult to predict accurately

Some host–guest complexes of the CB8 dataset proved to be more difficult to predict accurately compared to other such complexes in SAMPL8. This may not come as a surprise since CB8 guests were drug-like molecules with more rotational degrees of freedom compared to GDCC guests, thus more complex to model accurately. GDCC guests were more fragment-like and relatively rigid.

**Fig. 8:**
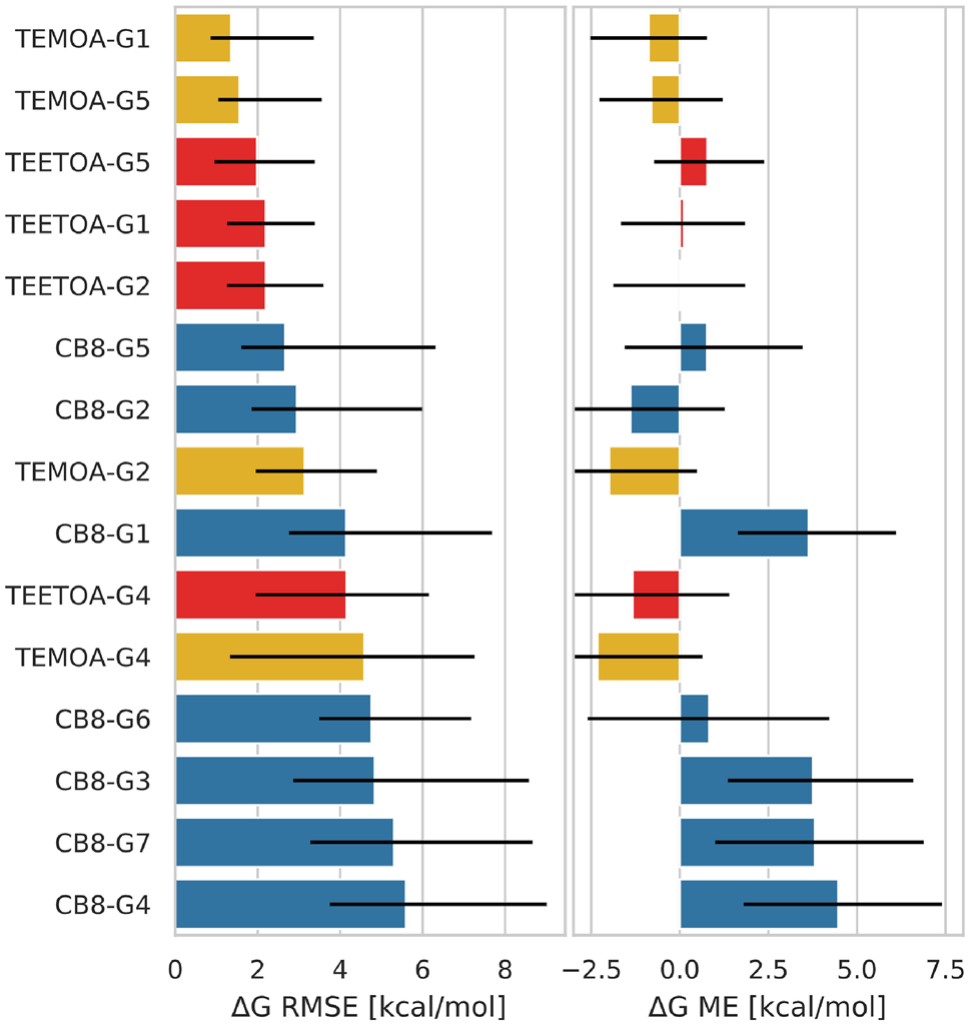
RMSE and ME statistics by host–guest system for ranked methods. Shown here are the binding free energy error metrics by host–guest system (CB8, TEMOA, and TEETOA) across ranked methods. The root mean square error (RMSE) and mean signed error (ME) for $\Delta G$ were computed via bootstrapping with replacement, which included experimental uncertainties, for all host–guest systems. This analysis includes all ranked methods submitted, but does not include predictions which were considered optional (CB8–G8, CB8–G9, and TEMOA–G3) in the analysis. The black error bars in the plots correspond to the 95-percentile bootstrap confidence intervals. Host–guest systems are color coded by host, where CB8 is represented by blue, TEMOA in yellow, and TEETOA in red

As shown in Fig. 8, there were 7 host–guest systems in SAMPL8 which had RMSE of about 4 kcal/mol or greater. Of these, indeed the majority of the molecules with the lowest accuracy (CB8–G4, CB8–G7, CB8–G3, CB8–G6, and CB8–G1) were in the CB8 dataset. The majority of methods tended to predict binding free energies for these systems to be less favorable, and the largest $\Delta \Delta G$ errors were more than 8 kcal/mol too favorable (Fig. S14). Guests G3 (morphine), G4 (hydromorphone), and G7 (cocaine) were the more complex guests in the SAMPL8 host–guest challenge, with the presence of multi-ring heterocycles at their core (Fig. 1), and had the largest errors for any host–guest complex in this challenge.

At the cores of guests G3, G4, and G7 are nitrogen centers which may function as chiral centers if protonated. In addition, there are some uncertainties on the protonation states for these guests when bound to CB8. It has been shown that protonation state of guests upon binding to cucurbiturils are modulated due to p*K*_a_shifts. Specifically, previous work found exceptions to the common assumption that cucurbiturils selectively bind protonated/cationic ammonium-based guests [64], and perhaps some SAMPL8 guests fit this exception. The majority of participants modeled the protonation states of the guests based on the p*K*_a_in solution, however, assuming a substantial p*K*_a_shift occurs upon binding, CB8 perhaps binds a different protomer of the guests, perhaps even in all cases.

For the GDCC dataset, binding free energy calculations had greater errors for guests G2 and G4 in the presence of the TEMOA host (Fig. 8). The guests differ modestly in their amphiphilic character compared to other GDCC guests, where guests G2 and G4 contain two polar edges with a hydrophobic center (Fig. 2). Thus, their interactions with the host and hydrating cavity waters and/or bulk solvent would differ. These characteristics for G2 and G4 would have greater modeling and simulation implications in the presence of TEMOA, where the binding mechanism is believed to involve the guest displacing cavity waters. Perhaps this water displacement poses particular modeling challenges, in terms of sampling or other issues, warranting further investigation. The higher predictive power of the AMOEBA/DDM/BAR method suggests polarization effects and change(s) in dipole moment of molecules in the cavity environment are a source of error(s).

On the other hand, binding free energy predictions for G1 and G5 with TEMOA were more accurate. G1 and G5 are more amphipathic, with a single polar end, a carboxylic acid, and the other end being strictly hydrophobic. The hydrophobic end is buried in the cavity interacting with the host. The carboxylic acid points away from the cavity and interacts with the bulk solvent and not with waters in the TEMOA cavity upon binding. Compared to G2 and G4, the amphiphilic character of G1 and G5 could explain the higher predictive accuracy for these systems even without explicitly modeling polarizing effects.

In contrast, binding free energy calculations had greater errors for guests G1 and G5 in the presence of the TEETOA host (Fig. 8). Meanwhile, between the two guests, errors were larger for G1 compared to G5. We speculate this may be due to the rearrangement of the ethyl groups at the cavity entrance. Particularly, we could expect the ethyl group rearrangement to be of greater importance for TEETOA–G1, since G1 is larger and would likely require (more of) the ethyl substituents to point away from the cavity for binding. However, this would be difficult to know without follow up studies of this particular system for methods used in SAMPL8.

#### Accuracy of predicting the tightest and weakest binders

In SAMPL8, methods were surveyed for their ability to correctly predict the tightest and weakest binders in each dataset. The tightest binders across host–guest datasets were CB8–G6, TEMOA–G2, and TEETOA–G2. As expected, methods that predict the tightest binders correctly are typically the top performing methods in each host–guest dataset (such as *DDM/FEP/MBAR/FM/RW[pm6s6]*, *DDM/FEP/MBAR/FM/[mp2,b3lyp]*, *DDM/AMOEBA/BAR*, and *ATM/GAFF2-AM1BCC/TIP3P/HREM*).

The weakest binders of SAMPL8 were CB8–G1, TEMOA–G3, and TEETOA–G5. For TEETOA, here we default to TEETOA–G5 as the weakest binder since TEETOA–G3 was not detected and it’s uncertain if this is a binder at all. No method predicted CB8–G1 or TEMOA–G3 correctly as the weakest binders for their respective datasets, while only two methods (*DDM/AMOEBA/BAR* and *ATM/GAFF2-AM1BCC/TIP3P/HREM*) predicted TEETOA–G5 correctly as the weakest binder. Overall, most methods do a better job predicting binding of tight binders but perform poorly for particularly weak binders.

Another example where methods have difficulties in recognizing weak binders is with TEETOA–G3. As discussed previously, there was no clear evidence of binding observed experimentally for TEETOA–G3 at the detection threshold via ITC or H NMR, indicating that the $\Delta G$ would be more positive than $-0.95$ kcal/mol. None of the ranked submissions predicted this correctly. In fact, the computed binding free energies for TEETOA–G3 were all too favorable. The computed $\Delta G$ ranged from $-1.42$ to $-7.17$ kcal/mol.

### All submission analysis: ranked and non-ranked including reference calculations

In general, participants submit predictions generated by methods from one of various categories. The options given for the method category are “alchemical”, “physical”, “mixed”, or “other”, with the last of these including a variety of other approaches including machine learning for example. Many methods used to generate free energy predictions were free energy methods based on statistical mechanics, and these could be divided into pathway-based and alchemical methods. These two categories of method should give equivalent answers, but that may not always be the case. It is important to ensure consistency of $\Delta G$ estimates between independent methods, to increase confidence in their implementation and drive progress in the field. Here, we survey and compare ranked methods, particularly on similar methods based on the same energy model for consistency in the computed $\Delta G$.

In the GDCC dataset, we cross compared the predictions of similar approaches. The *ATM/GAFF2-AM1BCC/TIP3P/HREM* and *PMF/GAFF2-AM1BCC/TIP3P/MD-US* methods used the same energy model and were intended (by the participants) for comparison to one another and for cross validation of the newer ATM method. The methods differ in that the ATM method is alchemical while the PMF method is path-based, and each utilizes a different sampling approach (see the literature [82] for more details). Both methods have similar accuracy in binding free energy predictions, with an RMS difference between these methods of 0.40 and 0.73 kcal/mol for TEMOA and TEETOA, respectively.

The top two methods, *DDM/AMOEA/BAR* and *ATM/GAFF2-AM1BCC/TIP3P/HREM*, made different predictions despite similar overall accuracy. The RMS difference between these methods was 1.79 kcal/mol for TEMOA and 2.30 kcal/mol for TEETOA. These methods used similar approaches but differ in the choice of force field (AMOEBA vs. GAFF2), and outliers between the methods highlight the current limitations and advantages of these models.

Comparing predictions of *APR/GAFF2-AM1BCC/TIP3P/US/TI* (reference calculations) with the top performing *PMF/GAFF2-AM1BCC/TIP3P/MD-US* method also using GAFF2, we observe an RMS difference of 2.97 and kcal/mol for TEMOA and 3.32 kcal/mol for TEETOA. As indicated by these RMS differences, the predictions of the two methods indeed vary more for TEETOA systems (for 4 of 5 guests, predictions differ by at least 2 kcal/mol). One way in which the methods differ is that in reference calculations conformational restraints were applied on the TEETOA host cavity, so the fact that these methods yield different results suggests a binding mechanism involving TEETOA conformational change(s), likely of the ethyl side chains. In addition, the methods differed in their computed binding free energies for TEMOA–G2 and TEMOA–G3 by more than 4 kcal/mol in each case, while the remaining systems (TEMOA–G1, TEMOA–G4, and TEMOA–G5) were all within 0.13 kcal/mol of one another. The discrepancy for TEMOA–G2 is likely due to modeling differences of G2, where the reference had a deprotonated (charged) hydroxyl while in the PMF approach both protonation states of the hydroxyl form were considered. The use of conformational restraints of TEMOA may play a role rendering G3 particularly sensitive to this, though the reason for the discrepancy between TEMOA–G3 predictions with these two methods is not obvious.

Many participants included additional binding free energy predictions as “non-ranked” submissions. For the most part, the difference between additional submissions is a single change such as using a different force field, a different sampling technique, or a different charging scheme. The submissions have also been analyzed and included in Table 3. Here we survey the sensitivity and impact of such changes on binding free energy predictions for the SAMPL8 host–guest challenge (for ranked and non-ranked methods).

One group provided a total of 13 different CB8 prediction sets based on MD free energy calculations with parameters from a FM protocol as previously applied in SAMPL6; some of their prediction sets, including their best ranked set of predictions, then used reweighting to re-evaluate free energies with a quantum mechanical energy function. The best approach (which was also the ranked approach) in this case used force-matched PM6-D3H4 parameters for the CB8 guests (*DDM/FEP/MBAR/FM/[pm6pm6]*), and yielded an RMS error of 2.46 kcal/mol. This suggests that hybrid approaches involving MD-based simulations with QM reweighting may now be able to achieve some measure of success.

Three different SILCS methods were applied to CB8 and achieved reasonable success; the top-performing method was a non-ranked submission which included empirical weighting factors applied to the computed grid free energies in order to improve agreement with experimental results for CB8 in SAMPL6. This empirical tuning resulted in better performance here than for the other two SILCS-based methods (with an overall RMS error of 1.96 kcal/mol), though this submission (*SILCS/LGFE/TIP3P/GCMC-MD/rew*) was not ranked. This seems to further illustrate that empirical corrections to computed binding free energies can improve accuracy, at least in some cases.

Aside from these cases, it has been difficult to trace differences in outcomes to single factors such as the choice of method or force field. In general, we encourage participants in future challenges to attempt to isolate the contributions of individual choices to their overall accuracy, either by coordinating with other participants or by the use of non-ranked submissions like these.

It’s worth briefly speculating as to why GDCC binding affinity predictions may be more accurate than those for CB8. We speculate that methods achieve greater predictive power for GDCC systems because the guests are typically more rigid and “simpler” as opposed to guests in the cucurbituril datasets in this challenge, though also the availability of empirical data from prior CB8 studies may be helpful to tune methods (as indeed several methods using empirical corrections saw improved performance here). Sampling of water displacement and rearrangement has been reported to be a separate issue as well, or possibly the origin of problems in CB8. The binding of guests to CB8 also involves water displacement, but it is possible that additional complications not explicitly accounted for (i.e. p*K*_a_shifts, protonation state modulation) contribute to the larger error in predictions.

#### Reference calculations and retrospective tests

In this section we compare the two sets of reference calculations, consider additional retrospective tests with reference calculations, and analyze the results. Overall, reference calculations performed at about the 50th percentile (Table 3), and gave similar performance as top methods by a few error metrics. The retrospective studies included modeling different protonation states of guests and examining their effect on binding free energy predictions for CB8. For the GDCC dataset, we ran tests to study the effects of side chain orientation and/or its sampling and guest position/sampling on predictions.

CB8 has been featured in several SAMPL iterations (SAMPL5, SAMPL6, and SAMPL8), and in each of these challenges, CB8 binding affinities have been more difficult to predict accurately compared to those for other host families. As discussed above, previous experimental work reported protonation state modulation of guests upon binding to CB8, thus we thought that this could play a role here for guests with multiple protonation states potentially accessible at pH 7.4. Indeed, when we modeled the guests in different protonation states compared to our initial predictions, the binding $\Delta G$ estimate changed significantly and in some cases by more than 2 kcal/mol (Table S1). In addition, for each of the guests [G1–G5, and G7 (see Table S1)], predicted binding free energies using one of the protonation states (neutral or protonated) were in agreement with experimental values, though the protonation state yielding best agreement varied by guest. These findings are in-line with previous literature results, and may warrant further attention from participants, since most participants did not account for possible protonation state changes for any guest other than G5.

Our reference calculations encountered particularly severe problems for some host–guest complexes. For example, the TEETOA–G1 prediction for reference calculations was unfavorable at 2.79 kcal/mol, whereas experimentally binding was favorable. In the analysis of the initial simulation the guest leaves the TEETOA pocket, due to the ethyl groups remaining oriented towards the cavity. We tested how the host conformation affected binding by restraining the host ethyl groups to keep them oriented towards the cavity, and found that in this conformation the predicted free energy was $14.87 \pm 0.39$ kcal/mol. Upon analysis of the new simulation, we observed the guest also leaving the TEETOA cavity, and a poor overlap profile for the attach phase was observed, similar to that of the initial simulation without restraining ethyl groups. In a separate simulation, we restrained all ethyl groups to point away from the cavity resulting in a predicted binding affinity of $-1.04 \pm 0.54$ kcal/mol. Restraining the ethyl groups in the outward orientation improved our overlap profile, guest G1 remained in the TEETOA cavity, improved agreement with experiment, and was in agreement with the similar PMF approach.

We also examined two different small-molecule force fields and observed similar performance. Particularly, we compared our two sets of reference calculations which differ only in the force field used (GAFF2 or OpenFF Parsley v1.2.0), and found that the force fields have similar performance (where on average predictions were within 0.5 kcal/mol of one another by comparing their predicted binding affinities). However, for certain systems (CB8–G5, CB8–G6, CB8–G7, TEMOA–G2, TEMOA–G4, TEETOA–G4, and TEETOA–G5) there is disagreement in the calculated values between GAFF2 and Parsley. The RMS difference between the two force fields were 3.00, 1.14, and 1.17 kcal/mol for CB8, TEMOA, and TEETOA, respectively.

#### Sensitivity of TEETOA host conformation to the guest orientation in the cavity

The GDCC TEETOA host has some degree of conformational flexibility which can be modulated by binding and by the guest orientation and identity. Thus, we performed additional calculations in which we applied the BLUES approach [100] to better understand the preferred orientations of the host’s four ethyl groups near the cavity opening. BLUES uses a hybrid of nonequilibrium candidate Monte Carlo (NCMC) and MD moves to enhance sampling of ligand binding modes for fragment-like small molecules in binding sites [100–102], rearrangements of receptor sidechains on ligand binding [103], rotation of internal torsions in ligands [104], and rearrangement of buried water molecules on ligand binding [105, 106]. More details of the approach can be found in prior work [100]. The BLUES package is freely available on GitHub at https://github.com/MobleyLab/blues. Here, we used BLUES moves to enhance sampling of the host ethyl groups in particular.

In our BLUES simulation, for each iteration, we randomly selected one of the four ethyl groups and applied a NCMC move. Instead of random angles, we biased our move proposals between predefined states of the ethyl group on the host. Specifically, a NCMC move was only proposed to either a state where the ethyl group pointed outward ($-150^{\circ }$ to $-50^{\circ }$) or inward (50$^{\circ }$ to 150$^{\circ }$), and only beginning from these states. This was the strategy we used in previous work [103] for more efficient sampling. In BLUES, each iteration was composed of a NCMC move and *m* MD steps (e.g., NCMC $\rightarrow$ MD $\rightarrow$ NCMC $\rightarrow$ MD). Since we focused NCMC moves on those two favorable states of the ethyl group, an NCMC move was only proposed if the current state fell within one of the two states. Otherwise, an additional *m* MD steps were performed. To ensure detailed balance, the ethyl group angle was evaluated after a NCMC move was executed so that the move was rejected if the resulting state fell outside of the two favorable states.

We started our simulations from a bound state TEETOA–G1 structure. We first minimized the system until forces were below a tolerance of 2.39 kcal/mol (10 kJ/mol by default via OpenMM) using the L-BFGS optimization algorithm [107]. Then 1 ns of NVT equilibration was performed at 298.15 K with all heavy atoms on the host and guest restrained (50 kcal/mol/Å$^{2}$). Long-range electrostatics were calculated using PME [108, 109] with nonbonded cutoffs of 10 Å. After that a series of NPT equilibration (2 ns for each) with decreasing restraints (a decrement of 5 kcal/mol/Å$^{2}$ in each run) were performed until the restraints were fully turned off. Then another 2 ns NPT run was performed without any restraints. The resulting conformation was confirmed as a bound state before the production phase.

We initialized BLUES simulations with five replicates. For each iteration, 1 NCMC move and 1000 MD steps were executed with hydrogen mass repartitioning scheme with 4 fs integration time step [110]. Each NCMC move was executed for 4400 steps (400 steps between lambda 0.0 and 0.2, 3600 steps between lambda 0.2 and 0.8, and 400 steps between lambda 0.8 to 1.0). This approach increased move acceptance in previous work [103].

3900 Iterations BLUES simulations were performed for each replicate. After checking collected data, we found (1) the ethyl groups orientations changed with the guest orientation in the pocket and (2) the guest unbound in in all 5 replicates. These results indicated the difficulty of adequate sampling of these ethyl groups and restraints were needed for both efficient sampling and keeping the host–guest in the bound state.

To seed more simulations, we clustered the trajectory where the host–guest was maintained in the bound state during the simulation using a distance based *k*-centers clustering method. The distance was computed between two carbon atoms (guest: C2, host: C9, Fig. S15A) that can represent different orientations of the guest and the distance between the guest and host sampled in the simulation. We picked four states of which three (States 1–3, Fig. S16) were the most populated and represent different orientations of the guest in the cavity. The remaining one (State 4, Fig. S16) was a conformation where the guest is right at the entrance of the host pocket. Position restraints with a spring constant of 20 kcal/mol/Å$^{2}$ were applied on the heavy atoms of the guest and two carbon atoms (C33 and C34, Fig. S15B) on the host. Three replicates of additional BLUES simulations were performed for each starting point and each replicate included 5000 iterations NCMC moves.

**Fig. 9:**
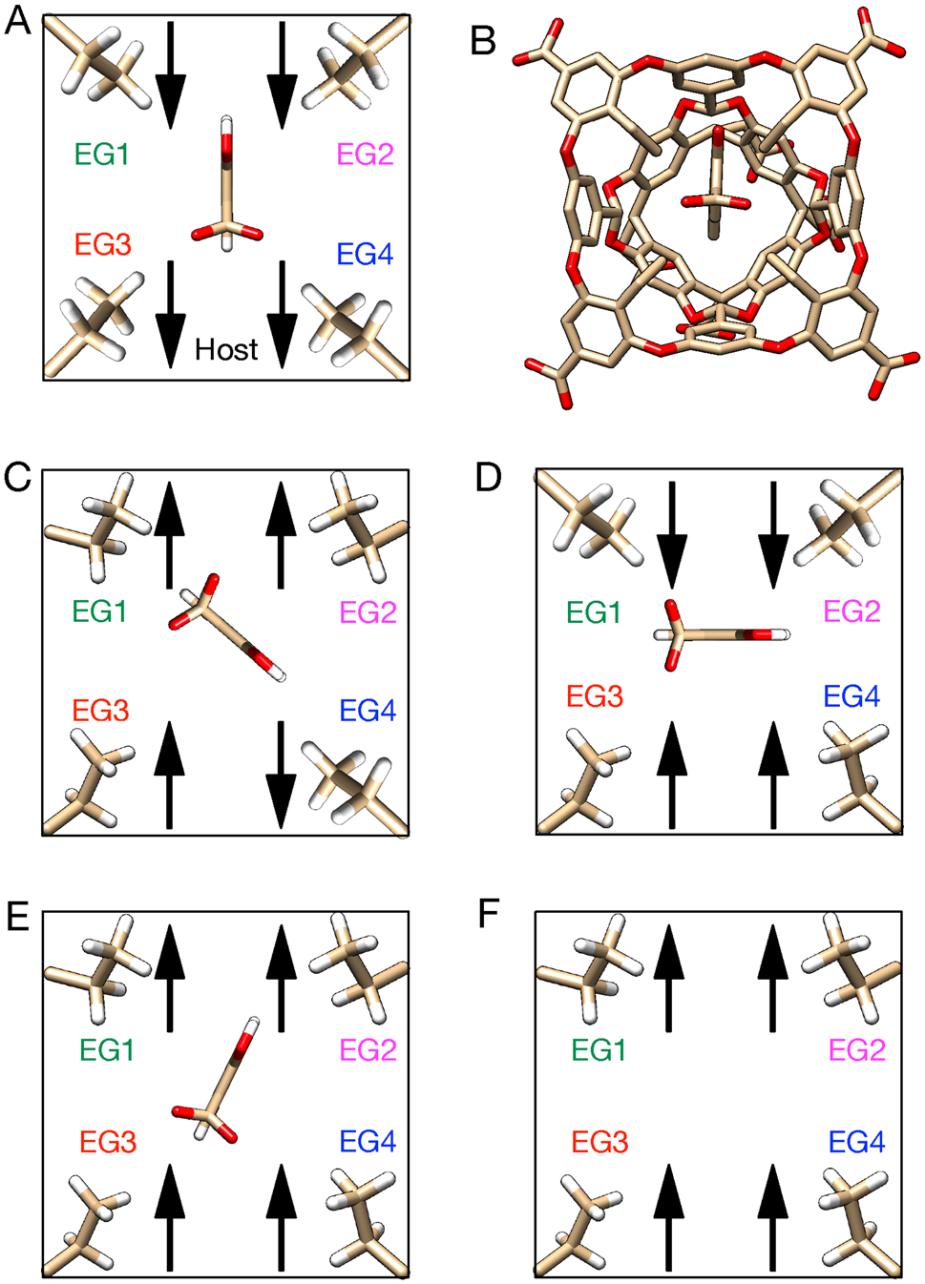
TEETOA ethyl group preferred orientations are dependent on the orientation of the guest. **A** A simplified cartoon of the host–guest system (TEETOA–G1), highlighting the guest and orientation of four ethyl groups (EG1–4). The host is represented by a square which has symmetry of four. **B** Shows the actual conformation of the host–guest system cartoon shown in **A**. **C**–**F** Cartoons show preferred orientations of the four ethyl groups with different guest orientations in the host pocket. The host orientation is the same as **A**. **A** Represents the orientations of the four ethyl groups in panel **B**. Arrows in **C**–**F** show preferred orientations of the four ethyl groups (up: outward, down: inward)

The additional BLUES simulations show that these four ethyl groups are very sensitive to the orientation of the guest in the cavity. Figure S17 shows the distribution of dihedral angles of the four ethyl groups sampled in BLUES simulations started from different conformations shown in Fig. S16. The position restraints on the guest and host ensure the orientation of the guest maintained the same in the context of simulations. Our results show that the ethyl groups’ preferred orientations are dependent on the orientation of the guest (Fig. 9; Fig. S17). For example, in Fig. 9, one ethyl group (EG4) prefers the inward orientation whereas the outward orientation is dominated in the other three ethyl groups (EG1–3). This guest orientation is also the most populated one from clustering of initial BLUES simulations. In Fig. 9B, we can see two ethyl groups (EG1–2) mainly point inward whereas the other two groups (EG3–4) always point outward in simulations. When the guest is at the entrance of the pocket (not bound yet), the four ethyl groups share a similar preference of orientation because of the symmetry of the host (Fig. 9D). Since the guest is not bound, more space is available in the pocket and all four ethyl groups can turn either inward or outward. The distribution of the four ethyl group orientations from simulations can be found in Fig. S17.

## Conclusions and lessons learned

The SAMPL8 host–guest challenge provided a platform to test the reliability and accuracy of current computational methods and tools to predict absolute binding free energies. As part of this challenge, hosts CB8 and TEMOA were revisited with new guest libraries, including a new host in the Gibb deep cavity cavitand family (of which the “octa-acid” host, common to several previous SAMPLs, is a member), TEETOA.

Similar to previous iterations of SAMPL, judging by the performance of submissions the CB8 systems posed a bigger challenge for participants. Five CB8 systems (CB8–G4, CB8–G7, CB8–G3, CB8–G6, and CB8–G1) all had an RMSE greater than 4 kcal/mol (Fig. 8; Fig. S14), compared to two in GDCC (TEMOA–G4 and TEETOA–G4). CB8 guests contained more complex fused ring systems at their nitrogen centers, and had additional protonation states that perhaps needed to be considered, likely complicating predictions.

The best ranked methods for CB8 were a free energy method based on force-matching (FM; *DDM/FEP/MBAR/FM/RW[pm6s6]*) followed by *SILCS/LGFE/TIP3P/GCMC-MD*. For this challenge, performance was variable with RMSE metric range from 2.43 to 6.64 kcal/mol, while correlation metrics for all methods were relatively poor. Only two methods achieved a coefficient of determination over 0.50. The few methods that achieved high correlation still had low binding free energy accuracy, which was a strong indicator of systematic errors. The FM method had a measure of success with its hybrid approach (MD-based with QM reweighting) method using force-matched PM6-D3H4 parameters for the CB8 guests.

When we consider all submissions (including non-ranked methods), a SILCS based approach (*SILCS/LGFE/TIP3P/GCMC-MD/rew*) had slightly better performance for the CB8 dataset. The SILCS based approach utilized an empirical approach, and illustrated that such corrections to binding free energy predictions did improve accuracy in many cases. (Several other SILCS-based methods did not perform as well.)

Experimental binding *enthalpy* values were also available in some cases, and the (*EE-MCC/GAFF2-AM1-BCC/TIP3P/MD/*) submission included predictions for these for the CB8 dataset, which were within 2 kcal/mol of experimental values for four of seven cases. Historically, accurate binding enthalpy/entropy predictions have been seldom seen, so this is exciting. However, since the challenge was based on predicting binding free energy, we must comment that binding free energy accuracy for this approach was low.

The *DDM/AMOEBA/BAR* method was the top performing method for the GDCC dataset (like in SAMPL7), followed by the *ATM/GAFF2-AM1BCC/TIP3P/HREM*. Although methods were generally more accurate on this dataset, there were some methods with limited predictive accuracy with RMSE values over 5 kcal/mol and coefficient of determinations as low as 0.01.

TEETOA is symmetrical and has four chemically equivalent ethyl groups. Based on our retrospective studies of TEETOA–G1, we conclude this symmetry is broken when G1 is bound because the guest (G1) has a particular orientation. So instead of all ethyl groups being oriented symmetrically, we find the preference is highly sensitive to the orientation of the guest. Our data suggests a specific orientation of the guest in the bound state (State 1 in Fig. S16) predominates. With this binding mode, the host has one ethyl group pointing inward whereas the other three point outward. Our results show details of the ethyl groups’ orientations and enhance our understanding of the likely bound conformation of TEETOA host–guest complexes. In addition, submissions for methods that used enhanced sampling techniques [Replica Exchange (RE) or REUS, GCMC] performed with greater accuracy than methods using classic MD or US techniques, showcasing the success and perhaps the necessity of enhanced sampling methods for adequately sampling host–guest bound conformations.

## Supplementary Information

Below is the link to the electronic supplementary material.

Supplementary file 1 (pdf 7554 KB)

## Data Availability

All SAMPL8 host–guest challenge instructions, submissions, experimental data and analysis are available at https://github.com/samplchallenges/SAMPL8/tree/master/host_guest. An archive copy of SAMPL8 GitHub repository host–guest challenge directory is also available in the Supplementary Documents bundle (*SAMPL8-supplementary-documents.tar.gz*). Some useful files from this repository are highlighted below. Table of participants submission filenames and their submission ID: https://github.com/samplchallenges/SAMPL8/tree/master/host_guest/Analysis/SAMPL8-user-map-HG.csv Submission files of prediction sets: https://github.com/samplchallenges/SAMPL8/tree/master/host_guest/Analysis/Submissions Python analysis scripts and outputs: https://github.com/samplchallenges/SAMPL8/tree/master/host_guest/Analysis/Scripts Table of performance statistics calculated for ranked methods for CB8 dataset: https://github.com/samplchallenges/SAMPL8/blob/master/host_guest/Analysis/Accuracy_ranked/CB8/StatisticsTables/statistics.csv Table of performance statistics calculated for all methods for CB8 dataset: https://github.com/samplchallenges/SAMPL8/blob/master/host_guest/Analysis/Reference/Accuracy/CB8/StatisticsTables/statistics.csv Table of performance statistics calculated for ranked methods for GDCC dataset: https://github.com/samplchallenges/SAMPL8/blob/master/host_guest/Analysis/Accuracy_ranked/GDCC_no_optional/StatisticsTables/statistics.csv Table of performance statistics calculated for all methods for GDCC (without optionals) dataset: https://github.com/samplchallenges/SAMPL8/blob/master/host_guest/Analysis/Reference/Accuracy/GDCC_no_optional/StatisticsTables/statistics.csv Table of performance statistics calculated for all methods for GDCC (with optionals) dataset: https://github.com/samplchallenges/SAMPL8/blob/master/host_guest/Analysis/Reference/Accuracy/GDCC/StatisticsTables/statistics.csv

## Abbreviations

SAMPL: Statistical Assessment of the Modeling of Proteins and Ligands
AM1-BCC: Austin model 1 bond charge correction
RESP: Restrained electrostatic potential
B3LYP: Beck 3-parameter Lee-Yang-Parr exchange-correlation functional [1, 2]
B3PW91: Becke 3-parameter Perdew-Wang 91 exchange–correlation functional [1]
GAFF: Generalized AMBER force field
CGenFF: CHARMM generalized force field
AMOEBA: Atomic multipole optimized energetics for biomolecular simulations [3]
DDM: Double decoupling method
DFT: Density functional theory
QM/MM: Mixed quantum mechanics and molecular mechanics
MMPBSA: Molecular mechanics Poisson–Boltzmann/solvent accessible surface area
MMGBSA: Molecular mechanics generalized born/solvent accessible surface area
TIP3P: Transferable interaction potential three-point
TIP4PEw: Transferable interaction potential four-point Ewald
p*K*_a_: −log$_{10}$ of the acid dissociation equilibrium constant
SEM: Standard error of the mean
RMSE: Root mean squared error
MAE: Mean absolute error
ME: Mean signed error
$\tau$: Kendall’s rank correlation coefficient (Tau)
R^2^: Coefficient of determination (R-Squared)
QM: Quantum Mechanics
MM: Molecular Mechanics
APR: Attach–Pull–Release
US: Umbrella Sampling
TI: Thermodynamic Integration
MBAR: Multistate Bennett Acceptance Ratio
FM: Force-Matching
