# Supplementary material for: An overview of the SAMPL8 host–guest binding challenge
Source: J Comput Aided Mol Des. 2022 Oct 14;36(10):707–34. doi: 10.1007/s10822-022-00462-5 (PMC9596595; doi:10.1007/s10822-022-00462-5)
Supplement: Supplementary file 1 — (pdf 7554 KB) [file 10822_2022_462_MOESM1_ESM.pdf]

9 Supplementary Information

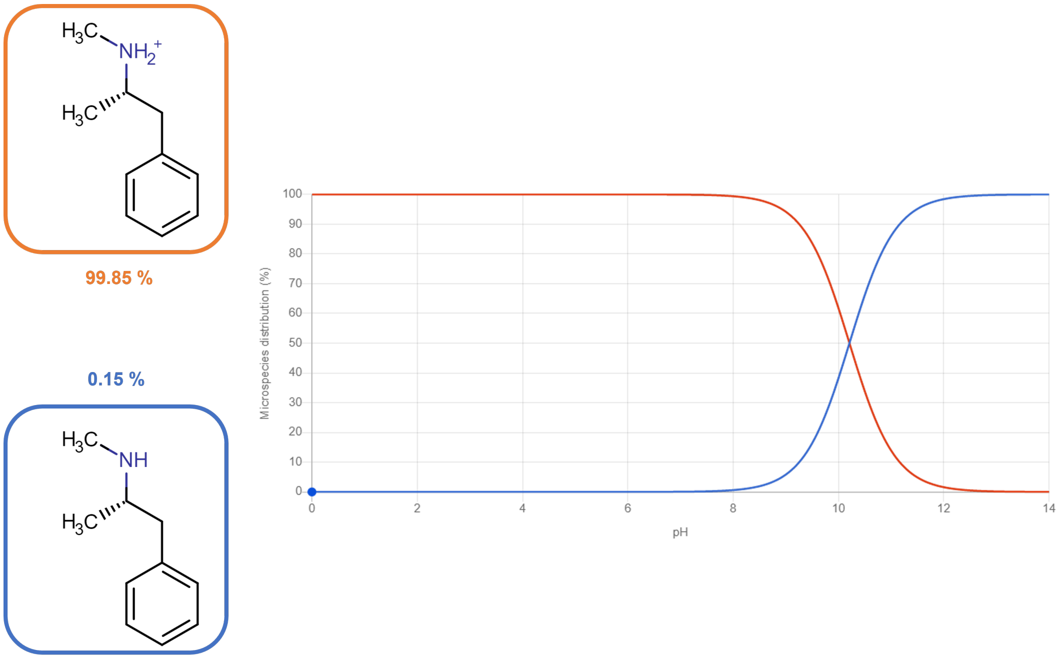

Figure S1. Microspecies distribution of protonation states for CB8-G1: Methamphetamine. Shown are the predicted microspecies distribution of protonation states for guest CB8-G1 from ChemAxon's Chemicalize. The structures of the microspecies are shown, and outlined with a box. The color of the box also represents the relative titration curve on the plot, showing the microspecies distribution as a function of pH. At the experimental pH of 7.4, the dominant microspecies of methamphetamine is with a protonated nitrogen (orange) having a 99.85% population. The second microspecies is the neutral variant (blue) having a 0.15% population.

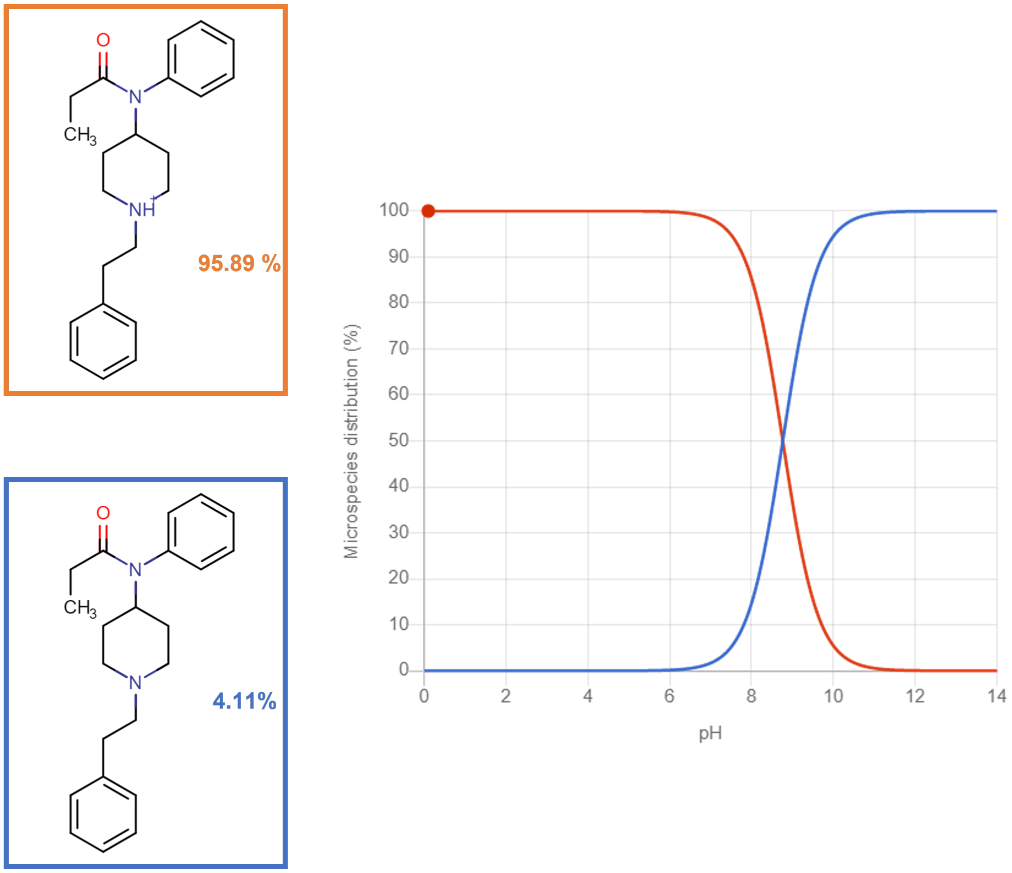

Figure S2. Microspecies distribution of protonation states for CB8-G2: Fentanyl. Shown are the predicted microspecies distribution of protonation states for guest CB8-G2 from ChemAxon's Chemicalize. The structures of the microspecies are shown, and outlined with a box. The color of the box also represents the relative titration curve on the plot, showing the microspecies distribution as a function of pH. At the experimental pH of 7.4, the dominant microspecies of fentanyl is with a protonated nitrogen (orange) having a 95.89% population. The second microspecies is the neutral variant (blue) having a 4.11% population.

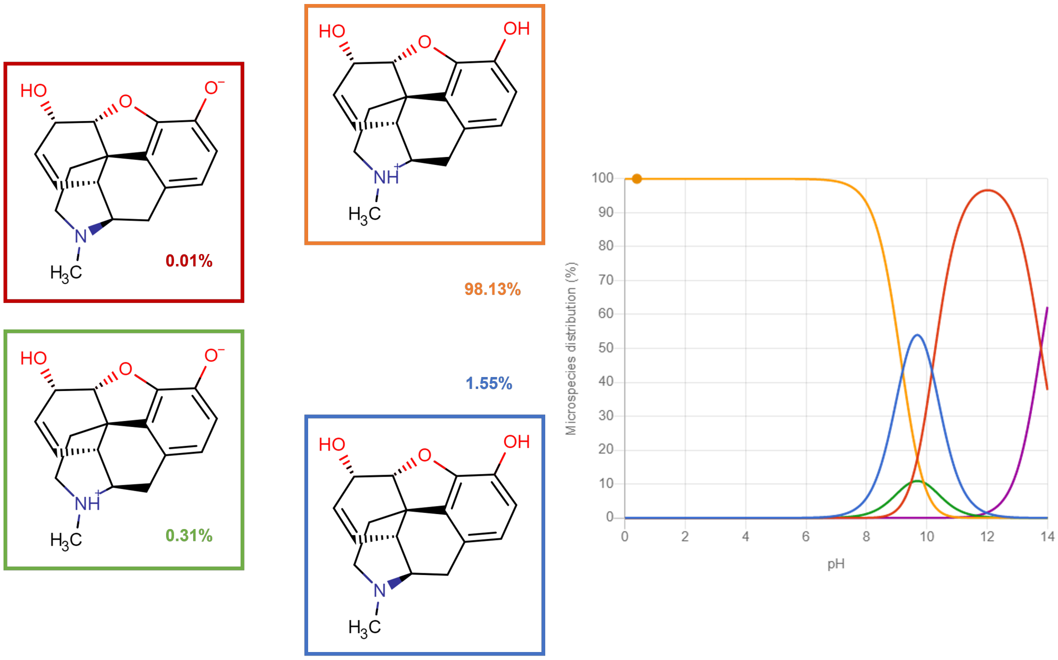

Figure S3. Microspecies distribution of protonation states for CB8-G3: Morphine. Shown are the predicted microspecies distribution of protonation states for guest CB8-G3 from ChemAxon's Chemicalize. The structures of the microspecies are shown, and outlined with a box. The color of the box also represents the relative titration curve on the plot, showing the microspecies distribution as a function of pH. At the experimental pH of 7.4, the dominant microspecies of morphine contains a protonated nitrogen (orange) having a 98.13% population. CB8-G3 has 3 additional microspecies, a neutral variant (blue), a zwitterionic like form (green), and a negatively charged variant (red) with one of its hydroxyls deprotonated. These microspecies are populated 1.55%, 0.31%, and 0.01% respectively.

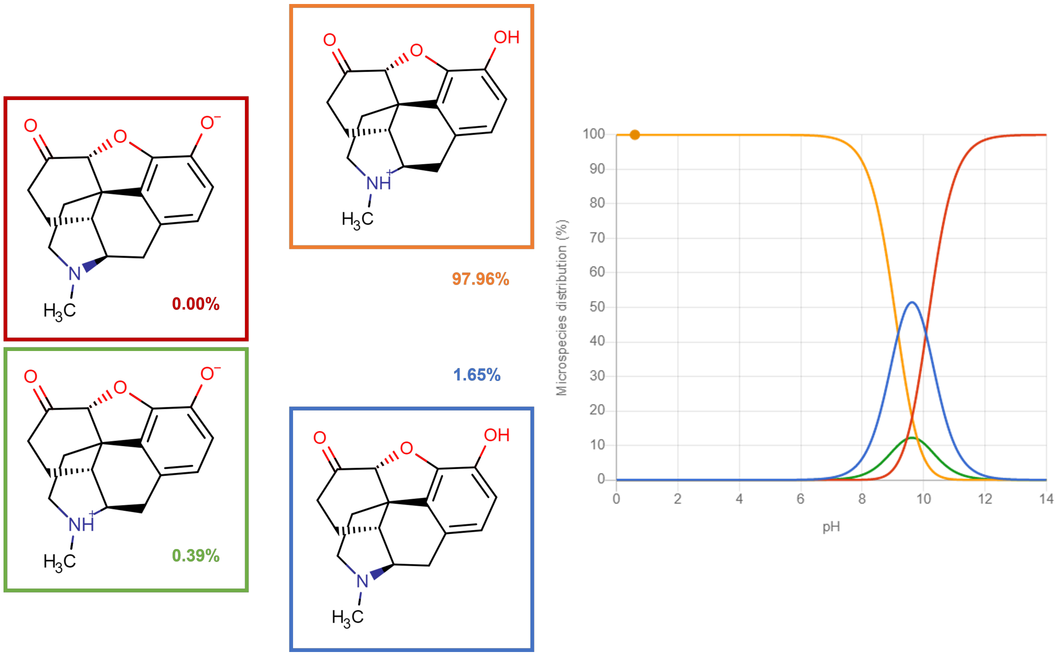

Figure S4. Microspecies distribution of protonation states for CB8-G4: Hydromorphone. Shown are the predicted microspecies distribution of protonation states for guest CB8-G4 from ChemAxon's Chemicalize. The structures of the microspecies are shown, and outlined with a box. The color of the box also represents the relative titration curve on the plot, showing the microspecies distribution as a function of pH. At the experimental pH of 7.4, the dominant microspecies of hydromorphone contains a protonated nitrogen (orange) having a 97.96% population. CB8-G4 has 3 additional microspecies, a neutral variant (blue), a zwitterionic like form (green), and a negatively charged variant (red) with one of its hydroxyls deprotonated. These microspecies are populated 1.65%, 0.39%, and 0.00% respectively.

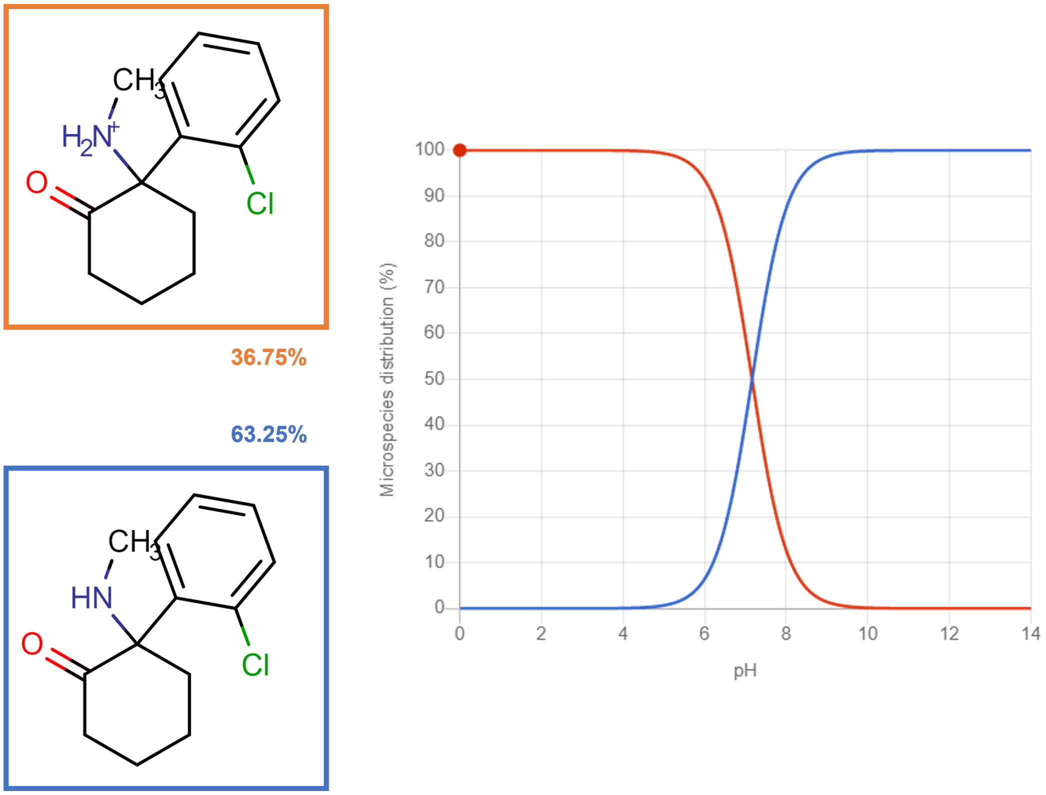

Figure S5. Microspecies distribution of protonation states for CB8-G5: Ketamine. Shown are the predicted microspecies distribution of protonation states for guest CB8-G5 from ChemAxon's Chemicalize. The structures of the microspecies are shown, and outlined with a box. The color of the box also represents the relative titration curve on the plot, showing the microspecies distribution as a function of pH. At the experimental pH of 7.4, the dominant microspecies of ketamine is with a neutral nitrogen (blue) having a 63.25% population. The second microspecies is the protonated and charged nitrogen variant (orange) having a 36.75% population.

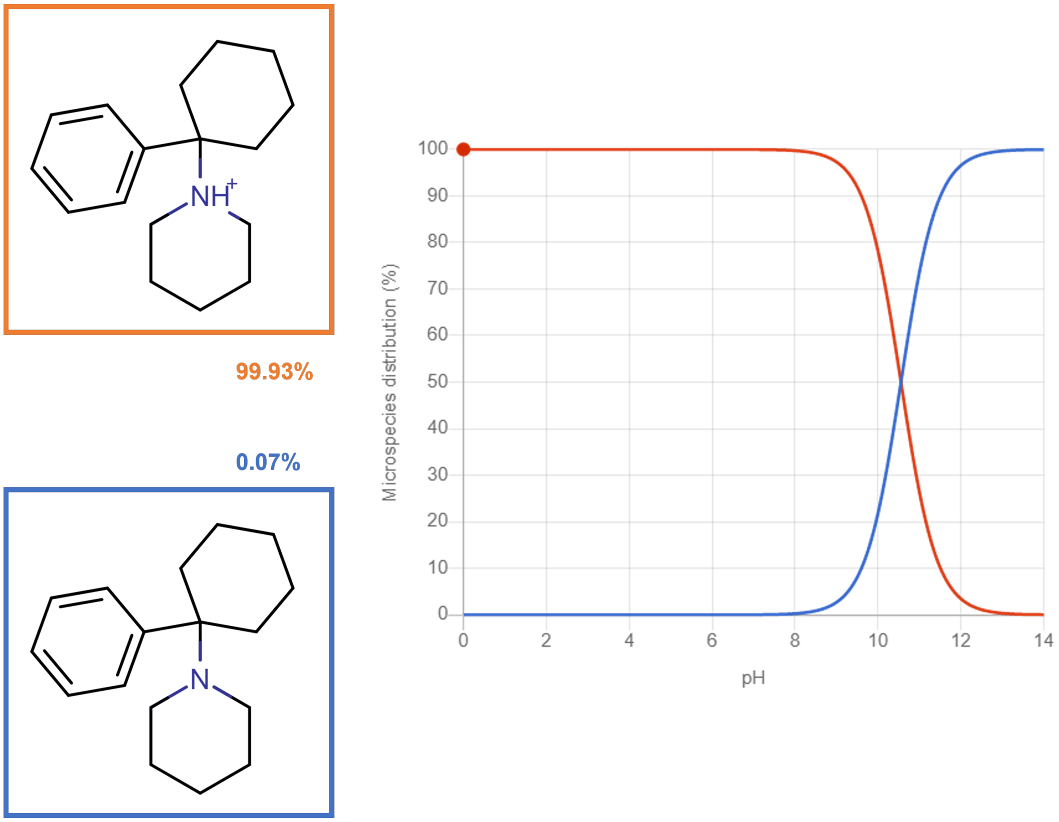

Figure S6. Microspecies distribution of protonation states for CB8-G6: PCP. Shown are the predicted microspecies distribution of protonation states for guest CB8-G6 from ChemAxon's Chemicalize. The structures of the microspecies are shown, and outlined with a box. The color of the box also represents the relative titration curve on the plot, showing the microspecies distribution as a function of pH. At the experimental pH of 7.4, the dominant microspecies of PCP contains a protonated and charged nitrogen (orange) having a 99.93% population. The second microspecies is with a neutral nitrogen variant (blue) having a 0.07% population.

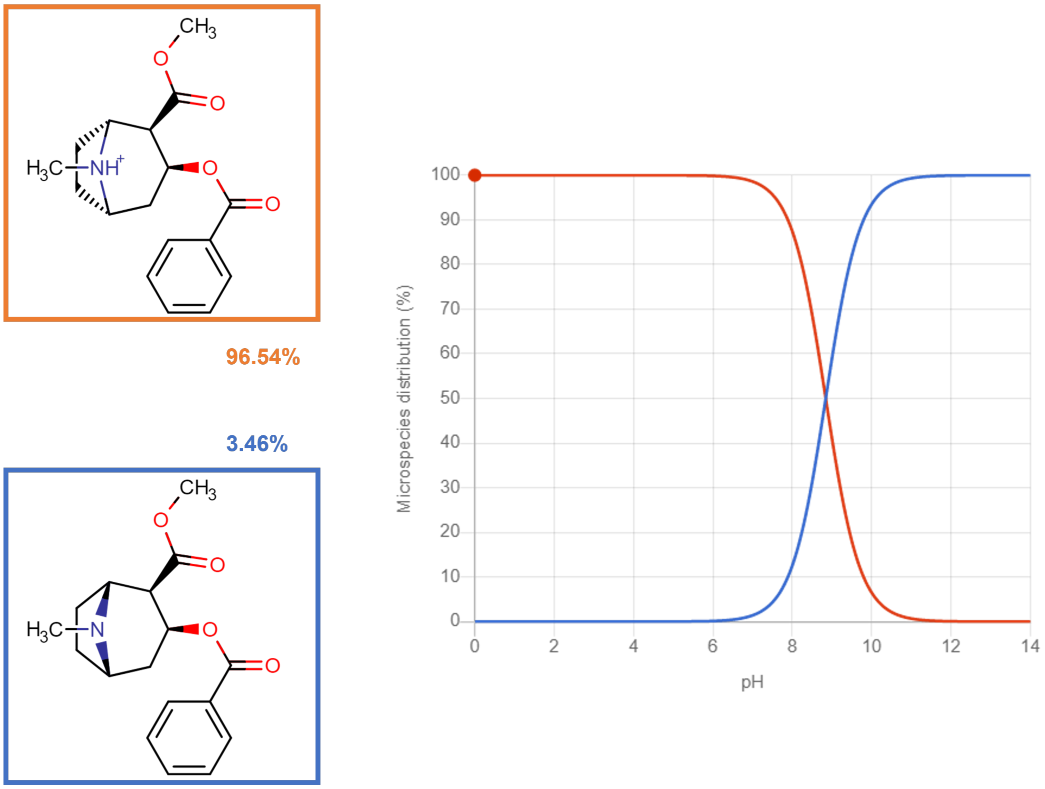

Figure S7. Microspecies distribution of protonation states for CB8-G7: Cocaine. Shown are the predicted microspecies distribution of protonation states for guest CB8-G7 from ChemAxon's Chemicalize. The structures of the microspecies are shown, and outlined with a box. The color of the box also represents the relative titration curve on the plot, showing the microspecies distribution as a function of pH. At the experimental pH of 7.4, the dominant microspecies of cocaine contains a protonated and charged nitrogen (orange) having a 96.54% population. The second microspecies is with a neutral nitrogen variant (blue) having a 3.46% population.

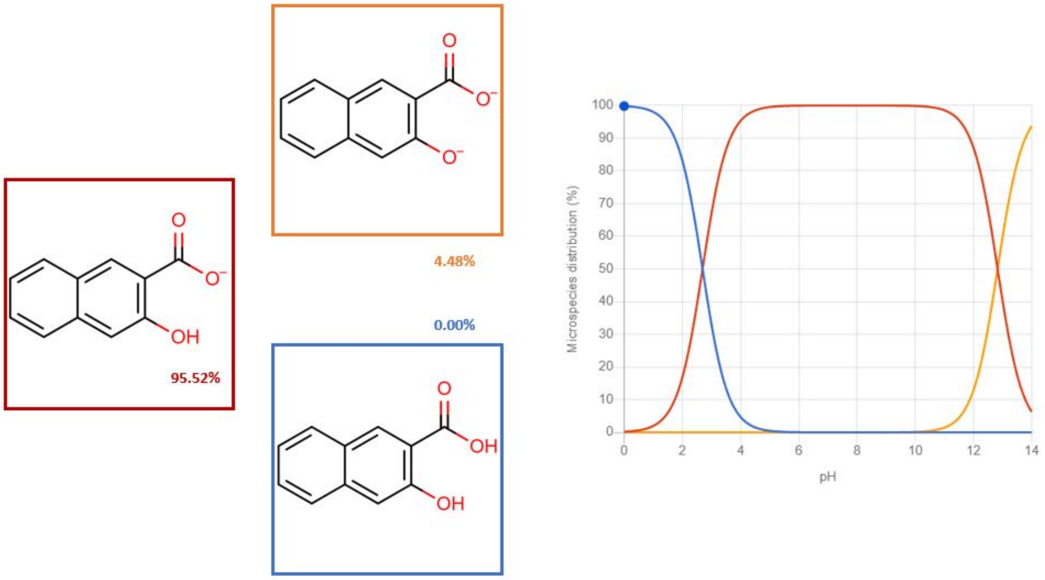

Figure S8. Microspecies distribution of protonation states for G1 in the GDCC dataset. Shown are the predicted microspecies distribution of protonation states for guest G1 from ChemAxon's Chemicalize. The structures of the microspecies are shown, and outlined with a box. The color of the box also represents the relative titration curve on the plot, showing the microspecies distribution as a function of pH. At the experimental pH of 7.4, the dominant microspecies of G1 contains a deprotonated and charged carboxylate oxygen (red) having a 95.52% population. A second microspecies is with a net -2 charge (orange) having a 4.48% population, and a neutral variant as the third microspecies (blue) that may not be populated.

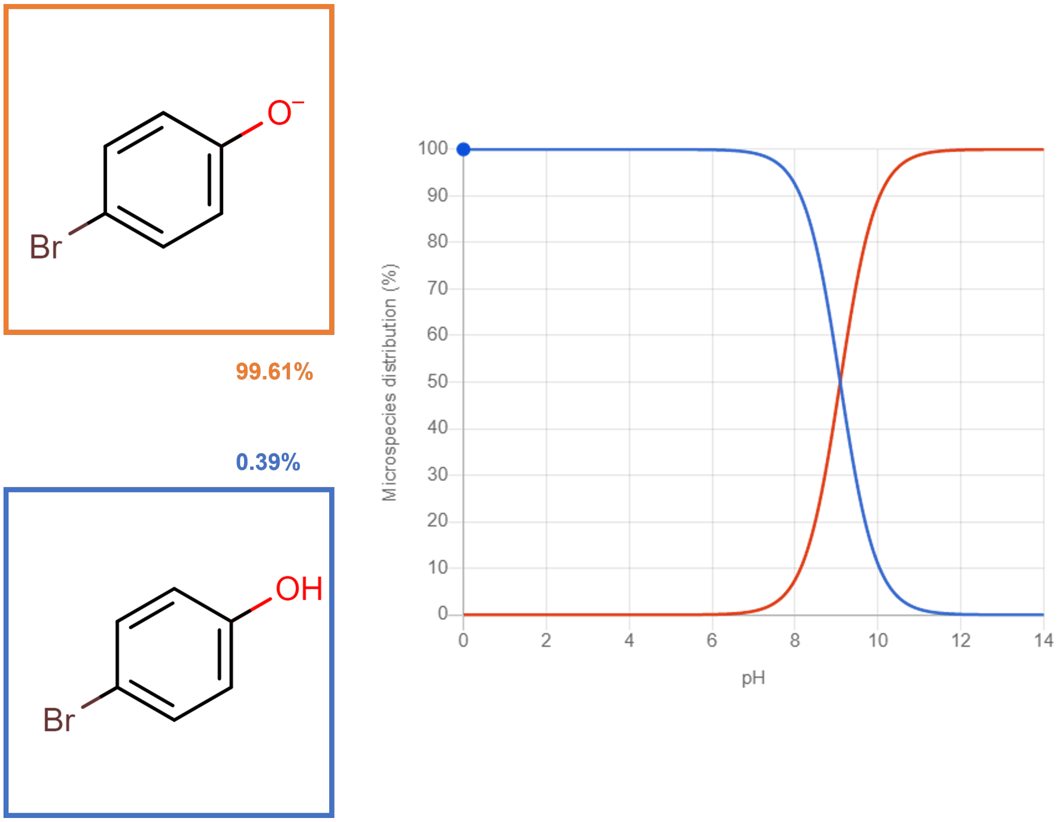

Figure S9. Microspecies distribution of protonation states for G2 in the GDCC dataset. Shown are the predicted microspecies distribution of protonation states for guest G2 from ChemAxon's Chemicalize. The structures of the microspecies are shown, and outlined with a box. The color of the box also represents the relative titration curve on the plot, showing the microspecies distribution as a function of pH. At the experimental pH of 7.4, the dominant microspecies of G2 contains a deprotonated and negatively charged hydroxyl oxygen (orange) having a 99.61% population. The second microspecies is with a neutral protonated hydroxyl oxygen (blue) having a 0.39% population.

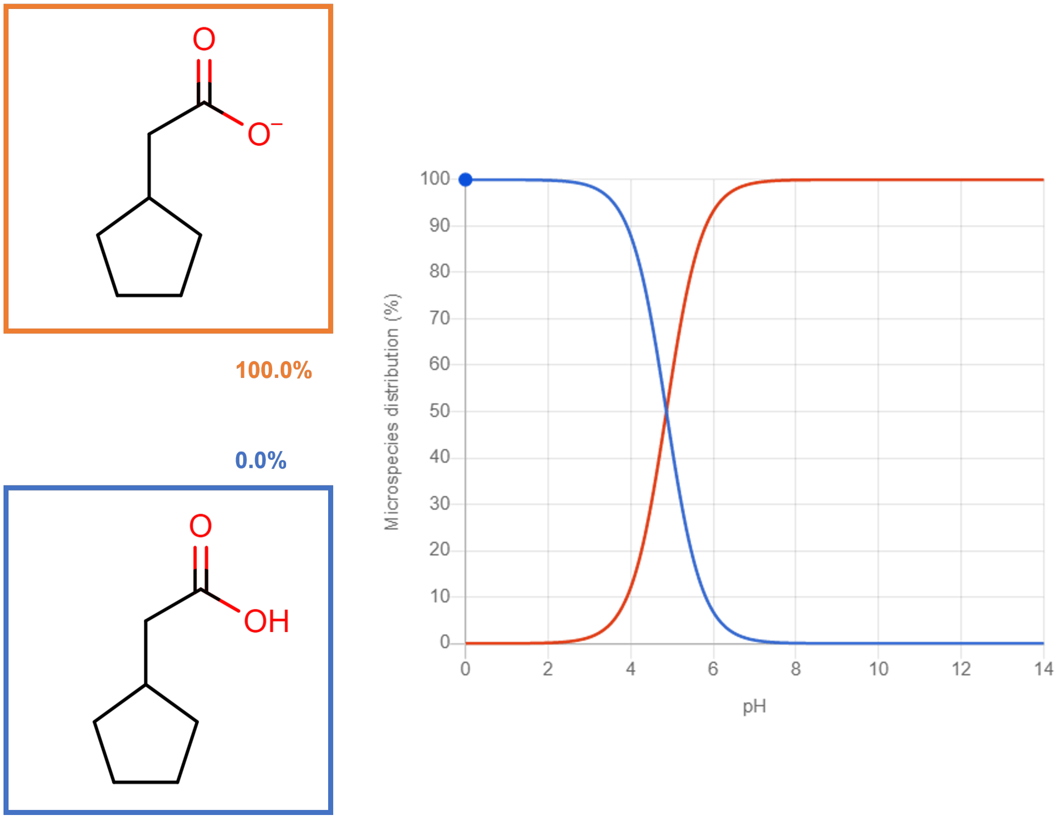

Figure S10. Microspecies distribution of protonation states for G3 in the GDCC dataset. Shown are the predicted microspecies distribution of protonation states for guest G3 from ChemAxon's Chemicalize. The structures of the microspecies are shown, and outlined with a box. The color of the box also represents the relative titration curve on the plot, showing the microspecies distribution as a function of pH. At the experimental pH of 7.4, the dominant microspecies of G3 contains a deprotonated and negatively charged carboxyl oxygen (orange) having a 100.0% population. The second microspecies is with a neutral protonated carboxyl oxygen (blue) having a 0.0% population.

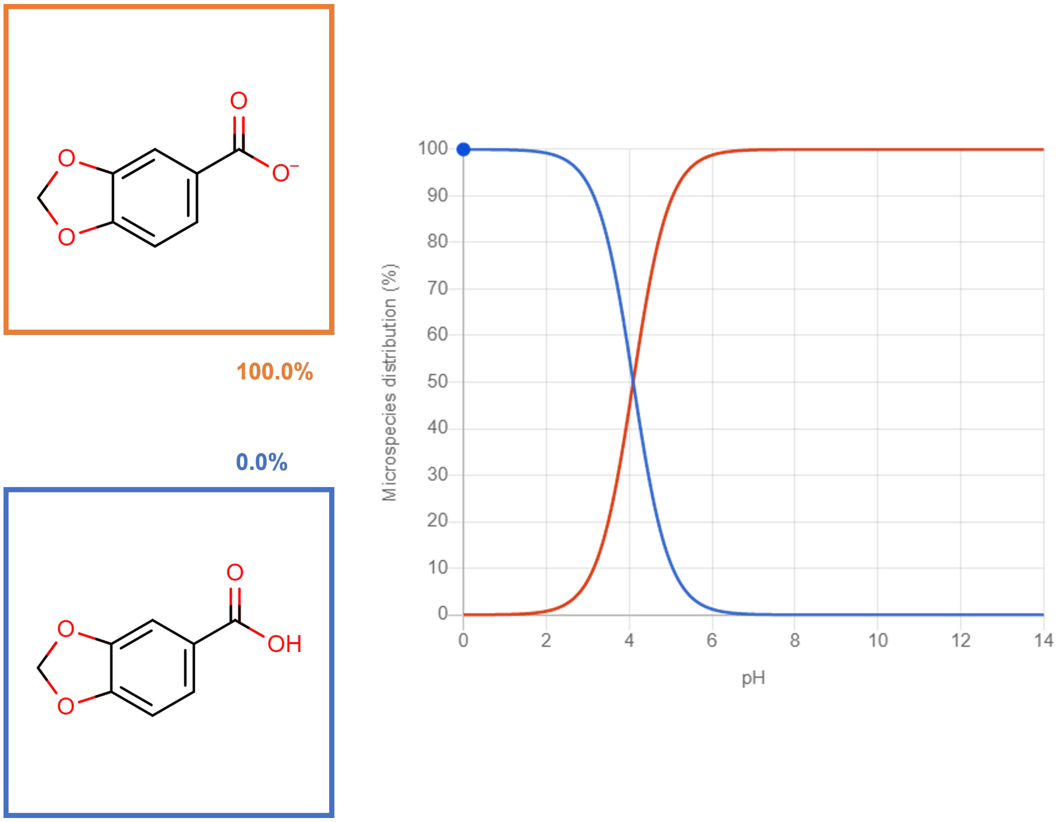

Figure S11. Microspecies distribution of protonation states for G4 in the GDCC dataset. Shown are the predicted microspecies distribution of protonation states for guest G4 from ChemAxon's Chemicalize. The structures of the microspecies are shown, and outlined with a box. The color of the box also represents the relative titration curve on the plot, showing the microspecies distribution as a function of pH. At the experimental pH of 7.4, the dominant microspecies of G4 contains a deprotonated and negatively charged carboxyl oxygen (orange) having a 100.0% population. The second microspecies is with a neutral protonated carboxyl oxygen (blue) having a 0.0% population.

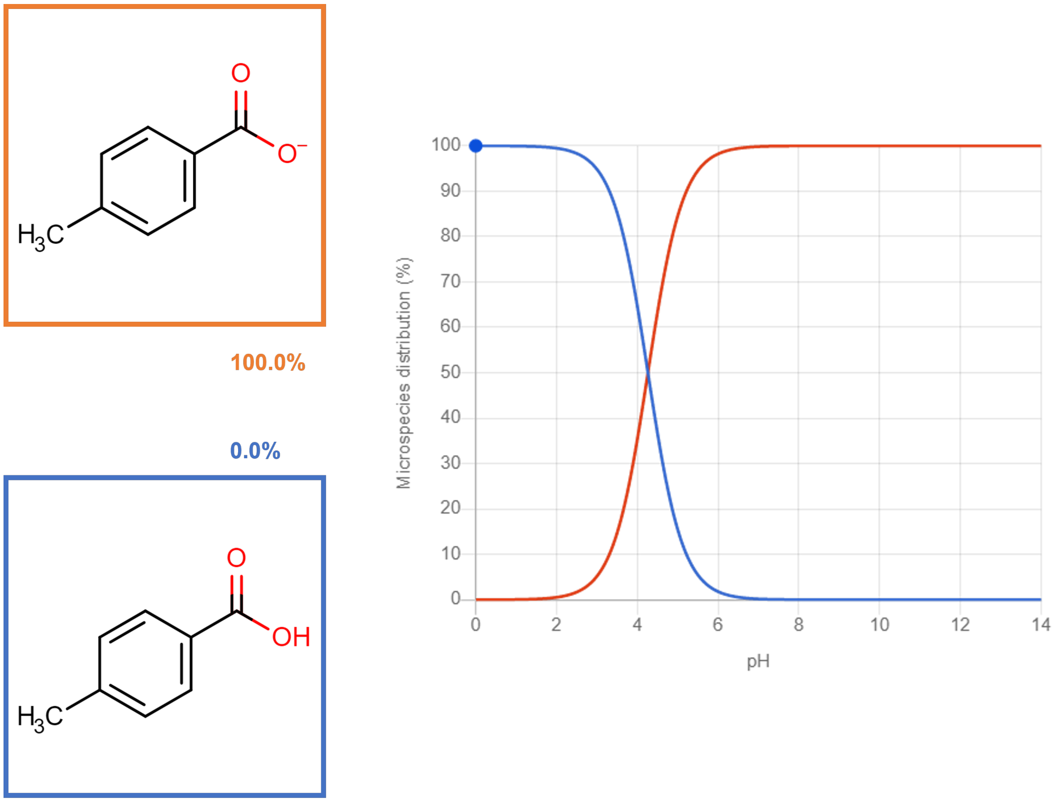

Figure S12. Microspecies distribution of protonation states for G5 in the GDCC dataset. Shown are the predicted microspecies distribution of protonation states for guest G5 from ChemAxon's Chemicalize. The structures of the microspecies are shown, and outlined with a box. The color of the box also represents the relative titration curve on the plot, showing the microspecies distribution as a function of pH. At the experimental pH of 7.4, the dominant microspecies of G5 contains a deprotonated and negatively charged carboxyl oxygen (orange) having a 100.0% population. The second microspecies is with a neutral protonated carboxyl oxygen (blue) having a 0.0% population.

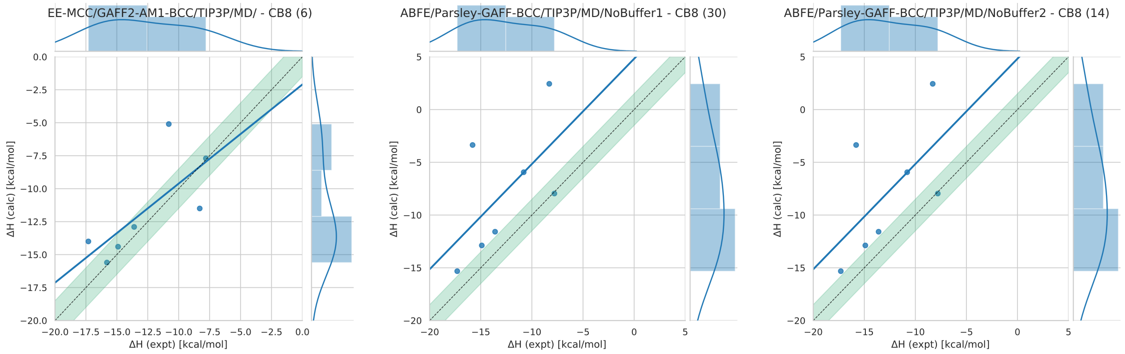

Figure S13. Correlation plots of Enthalpy predictions for the CB8 dataset. Shown here are correlation plots comparing calculated enthalpy values versus experimental for the *EE-MCC/GAFF2-AM1-BCC/TIP3P/MD*, *ABFE/Parsley-GAFF-BCC/TIP3P/MD/NoBuffer1*, and *ABFE/Parsley-GAFF-BCC/TIP3P/MD/NoBuffer2* predictions for the CB8 dataset. *EE-MCC/GAFF2-AM1-BC/TIP3P/MD* and *ABFE/Parsley-GAFF-BCC/TIP3P/MD/NoBuffer1* are ranked submissions, while *ABFE/Parsley-GAFF-BCC/TIP3P/MD/NoBuffer2* is a non-ranked submission.

Table S1. Summary of binding free energy reference calculations with GAFF2 force field for the SAMPL8 host-guest challenge using the Attach-Pull-Release (APR) Method. Table summarizes the calculated free energy and the standard error of the mean (SEM) for each phase (attach, pull, and release) of the APR method, the reference state free energy, and binding free energy (computed as the sum of all phases plus the reference state). In the System ID section, host-guest systems where multiple protonation states were considered include an additional letter P, N, D, or Z. A "P" denotes the guest with a protonated nitrogen with a +1 formal charge, a "N" denotes a guest with a neutral nitrogen, a "D" denotes a guest with a deprotonated hydroxyl oxygen with a -1 formal charge, and a "Z" denotes a guest as a zwitterion. TEETOA-G1-OUT and TEETOA-G1-IN represent retrospective calculations with harmonic restraints on the ethyl groups to orient them away (OUT) or towards (IN) the TEETOA cavity. All energies are reported in units of kcal/mol.

System ID	Attach FE	Attach SEM	Pull FE	Pull SEM	Release-host FE	Release-host SEM	Reference State- FE	Binding FE	Binding SEM
**GAFF2 - TI-Block**									
CB8-G1P	465.85	0.45	1.98	0.41	-447.92	0.22	-7.35	-12.56	0.65
CB8-G1N	462.65	0.63	1.53	0.27	-447.59	0.26	-7.35	-9.24	0.74
CB8-G2P	455.58	0.40	8.32	0.49	-447.85	0.23	-7.35	-8.40	0.68
CB8-G2N	450.76	0.60	6.01	0.30	-447.69	0.23	-7.35	-1.73	0.71
CB8-G3P	465.32	0.25	5.48	0.65	-447.53	0.24	-7.35	-15.91	0.74
CB8-G3D	453.94	0.42	2.02	0.54	-447.73	0.22	-7.35	-0.87	0.72
CB8-G3N	459.64	0.40	6.14	0.68	-447.60	0.22	-7.35	-10.83	0.82
CB8-G3Z	460.00	0.24	5.54	0.51	-447.44	0.27	-7.35	-10.74	0.62
CB8-G4P	460.95	0.23	10.97	0.35	-447.80	0.24	-7.35	-16.77	0.48
CB8-G4N	455.88	0.25	8.46	0.34	-447.63	0.29	-7.35	-9.36	0.51
CB8-G4D	452.40	0.39	4.71	0.35	-447.87	0.21	-7.35	-1.90	0.57
CB8-G4Z	457.71	0.42	7.74	0.69	-447.23	0.40	-7.35	-10.88	0.90
CB8-G5P	461.72	0.28	8.27	0.31	-447.44	0.24	-7.35	-15.90	0.49
CB8-G5N	459.25	0.41	8.22	0.35	-447.64	0.24	-7.35	-12.48	0.59
CB8-G6P	467.90	0.26	3.87	0.33	-447.37	0.23	-7.35	-17.06	0.48
CB8-G7P	457.91	0.56	4.75	0.57	-447.64	0.24	-7.35	-7.67	0.84
CB8-G7N	451.98	0.50	5.01	0.42	-447.48	0.22	-7.35	-2.16	0.69
TEMOA-G1	131.08	0.18	7.93	0.48	-125.33	0.19	-7.35	-6.33	0.55
TEMOA-G2D	130.20	0.14	7.75	0.28	-125.21	0.11	-7.35	-5.39	0.33
TEMOA-G3	130.06	0.15	6.10	0.31	-125.39	0.13	-7.39	-3.39	0.37
TEMOA-G4	131.82	0.13	9.52	0.31	-125.27	0.11	-7.35	-8.72	0.36
TEMOA-G5	130.76	0.11	9.97	0.32	-125.35	0.13	-7.35	-8.02	0.36
TEETOA-G1	123.54	0.28	7.83	0.26	-126.81	0.08	-7.35	2.79	0.29
TEETOA-G1-OUT	162.57	0.16	6.98	0.28	-162.77	0.12	-7.35	0.57	0.35
TEETOA-G1-IN	121.85	0.95	0.89	0.23	-127.76	0.07	-7.35	12.36	0.98
TEETOA-G2D	130.24	0.18	5.00	0.30	-126.82	0.08	-7.35	-1.07	0.36
TEETOA-G3	133.13	0.41	3.58	0.39	-126.89	0.11	-7.39	-2.44	0.57
TEETOA-G4	127.77	0.22	10.70	0.44	-126.63	0.15	-7.39	-4.45	0.51
TEETOA-G5	129.81	0.19	10.15	0.32	-126.91	0.11	-7.35	-5.70	0.38
**GAFF2 - MBAR**									
CB8-G1P	465.25	0.66	1.93	0.16	-448.93	0.65	-7.35	-10.91	0.94
CB8-G1N	463.65	0.85	1.52	0.17	-446.99	0.64	-7.35	-10.83	1.08
CB8-G2P	455.52	0.70	8.32	0.17	-448.12	0.71	-7.35	-8.38	1.01
CB8-G2N	451.07	0.70	5.99	0.17	-448.75	0.70	-7.35	-0.96	1.00
CB8-G3P	466.28	0.69	5.59	0.17	-447.11	0.57	-7.35	-17.41	0.91
CB8-G3N	454.76	0.70	20.50	0.17	-448.00	0.65	-7.35	-1.46	0.97
CB8-G3D	459.39	0.75	6.13	0.17	-448.25	0.71	-7.35	-9.92	1.04
CB8-G3Z	460.29	1.11	5.52	0.17	-447.88	0.73	-7.35	-10.57	1.34
CB8-G4P	461.19	0.78	10.95	0.17	-447.76	0.67	-7.35	-17.04	1.04
CB8-G4N	456.10	0.65	8.41	0.17	-447.34	0.77	-7.35	-9.83	1.02
CB8-G4D	452.34	0.86	4.70	0.17	-448.04	0.71	-7.35	-1.65	1.13
CB8-G4Z	457.35	0.95	7.75	0.24	-444.98	0.83	-7.35	-12.78	1.28
CB8-G5P	461.38	0.78	8.94	0.26	-447.33	0.77	-7.35	-15.65	1.11
CB8-G5N	459.58	0.78	8.22	0.17	-447.79	0.79	-7.35	-12.66	1.12
CB8-G6P	468.38	0.82	3.91	0.17	-447.76	0.76	-7.35	-17.17	1.14
CB8-G7P	457.18	0.65	4.69	0.17	-446.99	0.71	-7.35	-7.53	0.98
CB8-G7N	453.49	0.85	4.95	0.17	-446.78	0.61	-7.35	-4.31	1.06
TEMOA-G1	131.30	0.13	7.99	0.29	-125.51	0.12	-7.35	-6.43	0.34
TEMOA-G2D	130.32	0.09	7.67	0.28	-125.36	0.09	-7.35	-5.28	0.24
TEMOA-G3	130.21	0.09	6.06	0.20	-125.44	0.09	-7.39	-3.44	0.24
TEMOA-G4	131.88	0.09	9.53	0.21	-125.43	0.09	-7.35	-8.64	0.24
TEMOA-G5	130.88	0.09	9.95	0.21	-125.57	0.09	-7.35	-7.92	0.24
TEETOA-G1	123.71	0.06	7.81	0.15	-126.99	0.06	-7.35	2.81	0.17
TEETOA-G1-OUT	162.58	0.09	6.99	0.15	-162.80	0.09	-7.35	0.58	0.19
TEETOA-G1-IN	121.90	0.06	0.90	0.15	-127.87	0.06	-7.35	12.42	0.17
TEETOA-G2D	130.33	0.07	5.00	0.15	-127.02	0.06	-7.35	-0.96	0.17
TEETOA-G3	133.06	0.09	3.58	0.20	-127.01	0.09	-7.39	-2.25	0.24
TEETOA-G4	128.18	0.09	10.85	0.20	-126.82	0.09	-7.39	-4.83	0.24
TEETOA-G5	128.23	0.09	10.11	0.21	-127.01	0.09	-7.35	-3.99	0.24

Table S2. Summary of binding free energy reference calculations with Parsley 1.2.0 force field for the SAMPL8 host-guest challenge using the Attach-Pull-Release (APR) Method. Table summarizes the calculated free energy and the standard error of the mean (SEM) for each phase (attach, pull, and release) of the APR method, the reference state free energy, and binding free energy (computed as the sum of all phases plus the reference state). In the System ID section, host-guest systems where multiple protonation states were considered include an additional letter P, N, D, or Z. A "P" denotes the guest with a protonated nitrogen with a +1 formal charge, a "N" denotes a guest with a neutral nitrogen, a "D" denotes a guest with a deprotonated hydroxyl oxygen with a -1 formal charge, and a "Z" denotes a guest as a zwitterion. TEETOA-G1-OUT and TEETOA-G1-IN represent retrospective calculations with harmonic restraints on the ethyl groups to orient them away (OUT) or towards (IN) the TEETOA cavity. For some systems, reference calculations were not conducted and are labeled with NM. All energies are reported in units of kcal/mol.

System ID	Attach FE	Attach SEM	Pull FE	Pull SEM	Release-host FE	Release-host SEM	Reference State- FE	Binding FE	Binding SEM
**Parsley 1.2.0 - TI-Block**									
CB8-G1P	286.34	0.47	3.31	0.31	-270.98	0.20	-7.35	-11.32	0.60
CB8-G1N	NM	NM	NM	NM	NM	NM	NM	NM	NM
CB8-G2P	277.88	0.46	6.52	0.29	-270.71	0.22	-7.35	-6.34	0.59
CB8-G2N	281.38	0.57	6.98	0.44	-270.95	0.22	-7.35	-10.06	0.75
CB8-G3P	285.94	0.23	7.56	0.26	-270.74	0.22	-7.35	-15.41	0.41
CB8-G3N	285.98	0.27	8.64	0.74	-270.57	0.28	-7.35	-16.70	0.84
CB8-G3D	NM	NM	NM	NM	NM	NM	NM	NM	NM
CB8-G3Z	NM	NM	NM	NM	NM	NM	NM	NM	NM
CB8-G4P	284.57	0.26	9.31	0,69	-270.89	0.17	-7.35	-15.64	0.76
CB8-G4N	290.25	0.42	5.92	0.27	-270.86	0.21	-7.35	-17.96	0.54
CB8-G4D	NM	NM	NM	NM	NM	NM	NM	NM	NM
CB8-G4Z	NM	NM	NM	NM	NM	NM	NM	NM	NM
CB8-G5P	285.89	0.31	5.93	0.24	-270.96	0.24	-7.35	-13.51	0.46
CB8-G5N	284.66	0.71	5.75	0.41	-270.67	0.19	-7.35	-12.39	0.84
CB8-G6P	289.90	0.15	3.35	0.33	-271.05	0.31	-7.35	-14.85	0.48
CB8-G6N	287.61	0.36	4.07	0.48	-271.27	0.22	-7.35	-13.06	0.64
CB8-G7P	285.38	0.60	6.37	0.34	-270.79	0.29	-7.35	-13.61	0.75
CB8-G7N	289.90	0.46	1.45	0.38	-271.04	0.25	-7.35	-12.96	0.65
TEMOA-G1	142.47	0.18	7.88	0.43	-136.42	0.19	-7.35	-6.59	0.51
TEMOA-G2D	142.21	0.13	8.60	0.30	-136.28	0.11	-7.35	-7.17	0.34
TEMOA-G3	142.42	0.15	4.03	0.32	-136.21	0.13	-7.35	-2.89	0.37
TEMOA-G4	143.13	0.19	10.78	0.32	-136.15	0.12	-7.41	-10.36	0.39
TEMOA-G5	141.78	0.13	10.39	0.31	-136.26	0.14	-7.35	-8.57	0.37
TEETOA-G1	133.93	0.26	8.97	0.38	-137.51	0.10	-7.35	1.96	0.47
TEETOA-G1-OUT	136.28	0.10	7.56	0.25	-135.50	0.08	-7.35	-0.98	0.28
TEETOA-G1-IN	134.88	0.55	4.54	0.19	-138.52	0.08	-7.35	6.44	0.59
TEETOA-G2D	139.36	0.35	6.63	0.31	-137.39	0.09	-7.35	-1.25	0.47
TEETOA-G3	143.30	0.34	4.57	0.46	-137.58	0.22	-7.35	-2.95	0.61
TEETOA-G4	137.81	0.28	12.55	0.36	-137.35	0.10	-7.41	-5.59	0.46
TEETOA-G5	137.72	0.25	10.67	0.37	-137.48	0.11	-7.35	-3.57	0.47
**Parsley 1.2.0 - MBAR**									
CB8-G1P	286.53	0.07	3.31	0.05	-270.60	0.07	-7.35	-11.89	0.11
CB8-G1N	NM	NM	NM	NM	NM	NM	NM	NM	NM
CB8-G2P	277.36	0.07	6.52	0.05	-270.25	0.06	-7.35	-6.28	0.10
CB8-G2N	281.03	0.06	6.98	0.05	-270.61	0.06	-7.35	-10.10	0.10
CB8-G3P	285.42	0.08	7.55	0.05	-270.35	0.06	-7.35	-15.27	0.11
CB8-G3N	285.62	0.07	8.62	0.05	-270.26	0.06	-7.35	-16.63	0.10
CB8-G3D	NM	NM	NM	NM	NM	NM	NM	NM	NM
CB8-G3Z	NM	NM	NM	NM	NM	NM	NM	NM	NM
CB8-G4P	284.29	0.06	9.35	0.04	-270.57	0.05	-7.35	-15.72	0.09
CB8-G4N	289.62	0.08	5.79	0.05	-270.54	0.07	-7.35	-17.52	0.12
CB8-G4D	NM	NM	NM	NM	NM	NM	NM	NM	NM
CB8-G4Z	NM	NM	NM	NM	NM	NM	NM	NM	NM
CB8-G5P	285.99	0.07	5.94	0.05	-270.54	0.06	-7.35	-14.04	0.10
CB8-G5N	283.89	0.07	5.75	0.05	-270.30	0.06	-7.35	-11.99	0.10
CB8-G6P	289.43	0.08	3.41	0.05	-270.74	0.06	-7.35	-14.75	0.11
CB8-G6N	287.47	0.07	4.06	0.05	-270.96	0.06	-7.35	-13.22	0.10
CB8-G7P	285.19	0.07	6.34	0.05	-270.51	0.06	-7.35	-13.67	0.10
CB8-G7N	289.81	0.08	1.41	0.05	-270.67	0.07	-7.35	-13.20	0.12
TEMOA-G1	142.64	0.13	7.90	0.29	-136.66	0.13	-7.35	-6.53	0.35
TEMOA-G2D	142.28	0.09	8.58	0.21	-136.44	0.09	-7.35	-7.08	0.24
TEMOA-G3	142.52	0.10	4.01	0.21	-136.33	0.09	-7.35	-2.86	0.25
TEMOA-G4	143.14	0.10	10.79	0.20	-136.35	0.09	-7.41	-10.17	0.24
TEMOA-G5	141.94	0.09	10.38	0.21	-136.35	0.09	-7.35	-8.63	0.24
TEETOA-G1	133.99	0.07	8.94	0.17	-137.64	0.08	-7.35	2.06	0.20
TEETOA-G1-OUT	136.41	0.06	7.58	0.15	-135.60	0.06	-7.35	-1.04	0.17
TEETOA-G1-IN	NM	NM	NM	NM	NM	NM	NM	NM	NM
TEETOA-G2D	139.44	0.07	6.67	0.15	-137.55	0.07	-7.35	-1.21	0.17
TEETOA-G3	143.54	0.10	4.59	0.24	-137.74	0.13	-7.35	-3.04	0.29
TEETOA-G4	137.83	0.07	12.49	0.16	-137.51	0.08	-7.41	-5.40	0.19
TEETOA-G5	137.89	0.09	10.66	0.21	-137.60	0.09	-7.35	-3.61	0.24

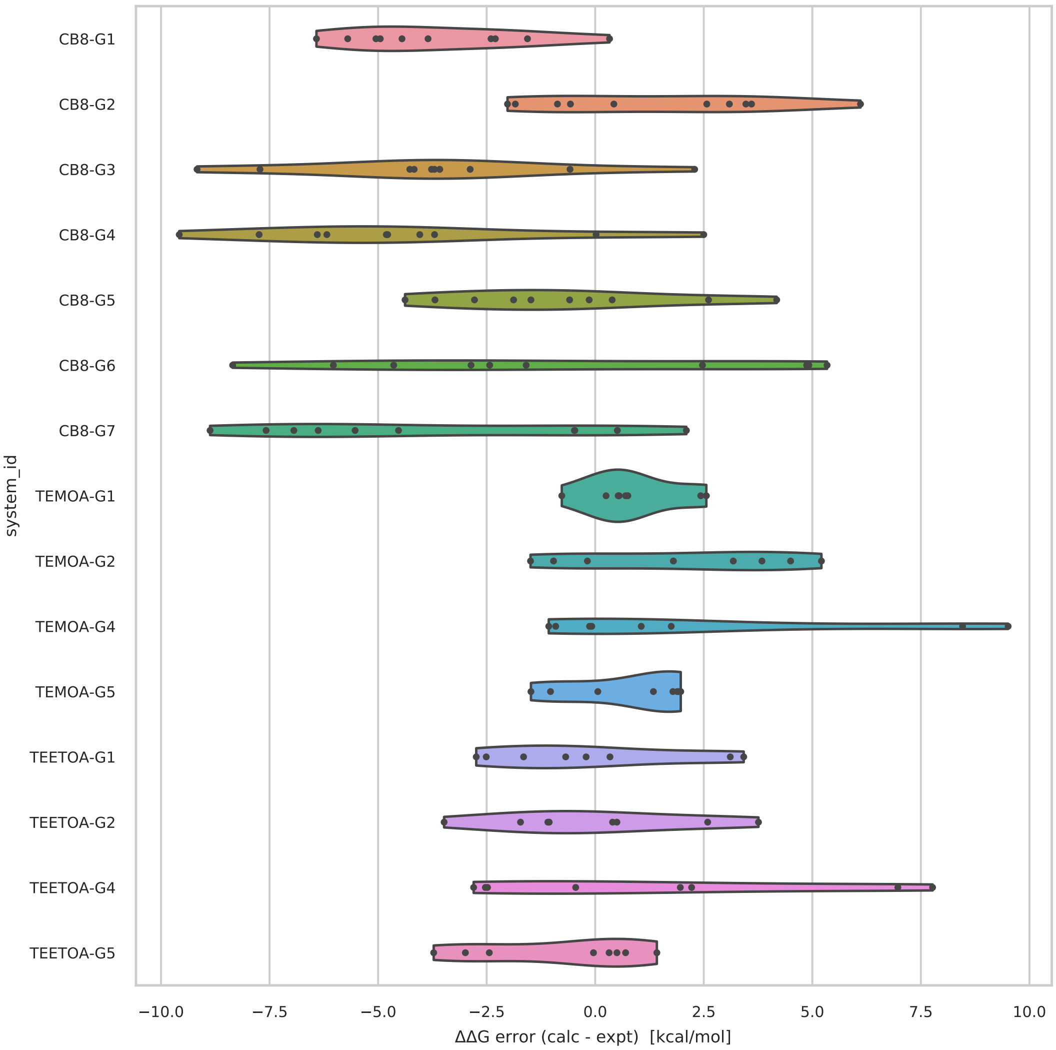

Figure S14. $\Delta\Delta G$ error of calculated versus experiment of all ranked submissions for each host-guest system. The $\Delta\Delta G$ error of calculated versus experiments of all ranked submissions for each host-guest system is shown. The $\Delta\Delta G$ error (in kcal/mol) of each prediction for a specific host-guest system is represented by a black dot. The distributions of the computed ΔΔ is highlighted around the individual points.

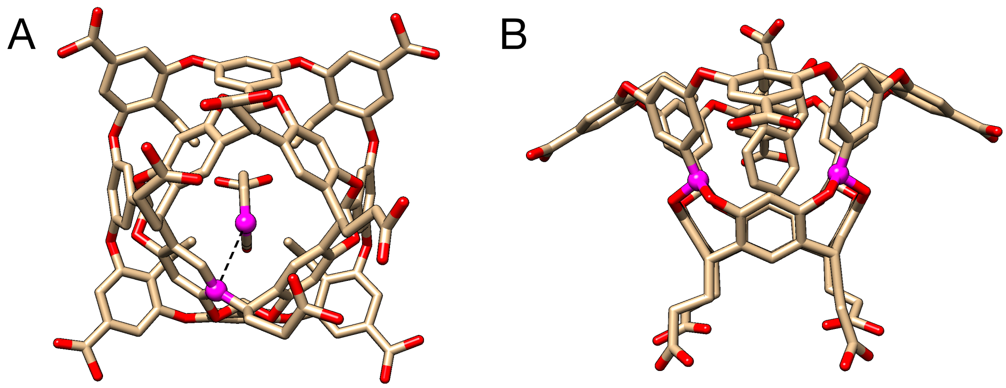

Figure S15. (A) The distance between two atoms on the host and guest (shown in magenta) was used in clustering of initial BLUES simulations. (B) Two atoms on the host (shown in magenta) along with all heavy atoms on the guest were used for position restraints.

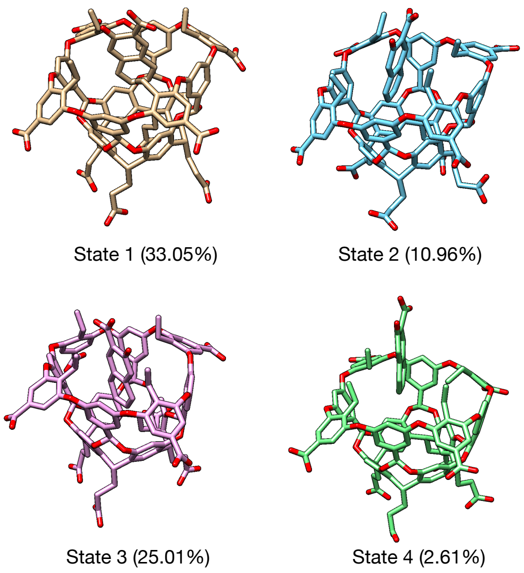

Figure S16. Four clustered states were picked for additional BLUES simulations with restraints. Three of them (State 1-3) are populated from clustering. State 4 is a unbound state where the guest is close to the entrance of the host. The state index and the corresponding populations from clustering are shown.

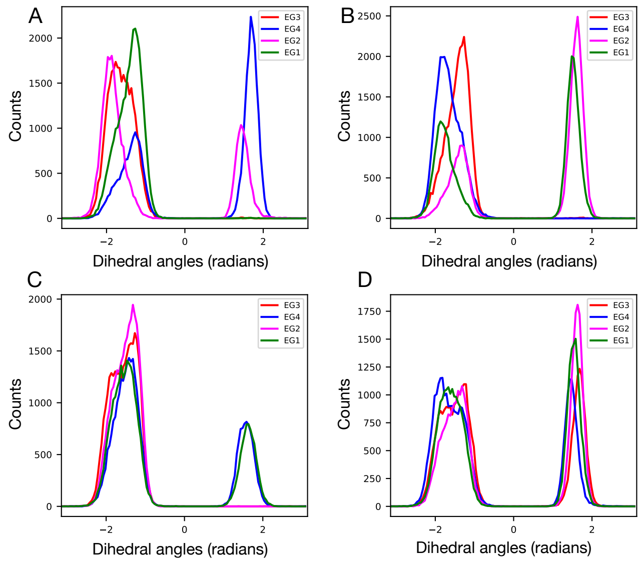

Figure S17. Distribution of dihedral angles of the four ethyl groups (EG1-4, Figure 9) sampled in BLUES simulations started from (A-D) State 1-4 shown in Figure S16. Positive and negative radians indicate the ethyl group point inward and outward, respectively.
